# CPT1A‐IL‐10‐mediated macrophage metabolic and phenotypic alterations ameliorate acute lung injury

**DOI:** 10.1002/ctm2.1785

**Published:** 2024-08-01

**Authors:** Muyun Wang, Di Wu, Ximing Liao, Haiyang Hu, Jing Gao, Linlin Meng, Feilong Wang, Wujian Xu, Shaoyong Gao, Jing Hua, Yuanyuan Wang, Qiang Li, Kun Wang, Wei Gao

**Affiliations:** ^1^ Department of Pulmonary and Critical Care Medicine Shanghai East Hospital, School of Medicine, Tongji University Shanghai China; ^2^ Department of Vascular Surgery Shanghai Sixth People's Hospital Affiliated to Shanghai Jiao Tong University School of Medicine Shanghai China; ^3^ Second Department of Respiratory and Critical Care Medicine The Fourth People's Hospital of Jinan Shandong China

**Keywords:** acute lung injury, carnitine palmitoyltransferase 1A, fatty acid oxidation, interleukin‐10, macrophage

## Abstract

**Background:**

Acute lung injury (ALI)/acute respiratory distress syndrome (ARDS) is a common acute respiratory failure due to diffuse pulmonary inflammation and oedema. Elaborate regulation of macrophage activation is essential for managing this inflammatory process and maintaining tissue homeostasis. In the past decades, metabolic reprogramming of macrophages has emerged as a predominant role in modulating their biology and function. Here, we observed reduced expression of carnitine palmitoyltransferase 1A (CPT1A), a key rate‐limiting enzyme of fatty acid oxidation (FAO), in macrophages of lipopolysaccharide (LPS)‐induced ALI mouse model. We assume that CPT1A and its regulated FAO is involved in the regulation of macrophage polarization, which could be positive regulated by interleukin‐10 (IL‐10).

**Methods:**

After nasal inhalation rIL‐10 and/or LPS, wild type (WT), *IL‐10* ^‐/‐^ , Cre^‐^ *CPT1A* ^fl/fl^ and Cre^+^ *CPT1A* ^fl/fl^ mice were sacrificed to harvest bronchoalveolar lavage fluid, blood serum and lungs to examine cell infiltration, cytokine production, lung injury severity and IHC. Bone marrow‐derived macrophages (BMDMs) were extracted from mice and stimulated by exogenous rIL‐10 and/or LPS. The qRT‐PCR, Seahorse XFe96 and FAO metabolite related kits were used to test the glycolysis and FAO level in BMDMs. Immunoblotting assay, confocal microscopy and fluorescence microplate were used to test macrophage polarization as well as mitochondrial structure and function damage.

**Results:**

In in vivo experiments, we found that mice lacking CPT1A or IL‐10 produced an aggravate inflammatory response to LPS stimulation. However, the addition of rIL‐10 could alleviate the pulmonary inflammation in mice effectively. IHC results showed that IL‐10 expression in lung macrophage decreased dramatically in Cre^+^ *CPT1A* ^fl/fl^ mice. The in vitro experiments showed Cre^+^ *CPT1A* ^fl/fl^ and *IL‐10* ^‐/‐^ BMDMs became more “glycolytic”, but less “FAO” when subjected to external attacks. However, the supplementation of rIL‐10 into macrophages showed reverse effect. CPT1A and IL‐10 can drive the polarization of BMDM from M1 phenotype to M2 phenotype, and CPT1A‐IL‐10 axis is also involved in the process of maintaining mitochondrial homeostasis.

**Conclusions:**

CPT1A modulated metabolic reprogramming and polarisation of macrophage under LPS stimulation. The protective effects of CPT1A may be partly attributed to the induction of IL‐10/IL‐10 receptor expression.

## Graphical abstract

- CPT1A modulated metabolic alteration and polarisation of macrophage under LPS stimulation.
- The protective effects of CPT1A may be partly attributed to the induction of interleukin‐10/interleukin‐10 receptor expression.
- The CPT1A‐IL‐10 axis and its regulated macrophage immunometabolism may provide a novel potential therapeutic targeting of ALI/ARDS.

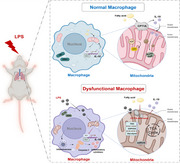

## 1 INTRODUCTION

Acute lung injury (ALI)/acute respiratory distress syndrome (ARDS) is a common acute respiratory failure due to diffuse pulmonary inflammation and oedema. ALI/ARDS is common in clinical syndrome, identified in 10.4% of intensive care unit admissions.^1^ The pathophysiology of ALI/ARDS is intricate, involving the misregulation of numerous superposition and interacting pathways of inflammation, coagulation, as well as injury, resulting in limited pharmacological treatment options and an overall mortality rate of 43%.^2^ One of the key immune cells in the lung, macrophages, plays a key regulatory role in the early inflammatory response and progression of ALI/ARDS.^3^ Therefore, a deeper understanding of the altered functional phenotypes and cellular processes of macrophages in ALI/ARDS pathology may lead to novel therapeutic strategies.

During the past few years, a number of academic achievements have emerged, emphasising the crucial function of metabolic remodelling in the phenotypic adjustment and plasticity of macrophages.^4^ Though the appearance of contradictory observations is emerging recently. In general, LPS or microbial stimuli‐activated proinflammatory macrophages (classically activated M1) typically exhibit increased glycolytic processes, impairment of mitochondrial oxidative phosphorylation (OXPHOS), disrupted tricarboxylic acid (TCA) cycle and overproduction of reactive oxygen species (ROS), along with elaboration of proinflammatory cytokines and increased microbial killing.^5^, ^6^, ^7^ When comparing the metabolic differences between M1 macrophages and anti‐inflammatory macrophages (alternatively activated M2), we found that the latter exhibited a complete citric acid cycle (TCA cycle) and a more active mitochondrial OXPHOS process, which are key features of their anti‐inflammatory phenotype.^8^

Another source of ATP supply in mitochondria is the oxidation of fatty acids, which is particularly important when glucose is underutilised. In addition to the field of bioenergetics,^9^ fatty acid oxidation (FAO) has been reported to facilitate the transition of macrophages to the M2 phenotype since 2006.^10^ It is now widely accepted that interleukin‐4‐rich macrophage differentiation (referred to as M2 polarisation in this study) is associated with elevated levels of FAO.^11^, ^12^ The evidence for confirmation comes primarily from the investigation of etomoxir, which has been shown to be effective as a carnitine acyltransferase 1 (CPT1) inhibitor.^13^, ^14^ CPT1, a rate‐limiting enzyme that plays an integral role in the FAO pathway, resides in the outer mitochondrial membrane. Its main function is to complex long‐chain fatty acid acyl coenzyme A (CoA) with carnitine, which in turn promotes the transport of this substance into mitochondria, where fatty acids undergo oxidation.^15^ CPT1A, an active form of the CPT1 system, is deeply involved in the biology of many lung diseases, such as ALI, chronic obstructive pulmonary disease, and bronchial asthma.^16^ However, the role of CPT1A and its‐mediated FAO in the regulation of macrophage phenotype during ALI/ARDS has not yet been thoroughly dissected.

Interleukin‐10 (IL‐10), a representative of regulatory cytokines, shows critical role in regulating innate as well as adaptive immune responses. It aims to reduce tissue damage caused by uncontrolled inflammation by suppressing the immune response.^17^ The generation of IL‐10 by myeloid cells (e.g., macrophages and dendritic cells) is primarily driven by a series of pattern recognition receptor (PRRS) signals, which including ligands for Toll‐like receptors (TLRs) and inflammation promoting factors.^18^ Recent findings have revealed that major metabolic regulatory turn points within macrophages can be activated in response to adjustments in nutritional and oxygen status, as well as in the signalling pathways of cytokine receptors such as IL‐10.^19^ Ip et al.^20^ revealed that lipopolysaccharide (LPS) induces the metabolic transition of macrophages to the glycolytic pathway, and at the same time, cytokine IL‐10 can neutralise this effect and catalyse the positive progress of OXPHOS. However, whether these metabolic switches, especially changes in FAO participant in the anti‐inflammatory capability of IL‐10 in the development of ALI, remained largely unknown.

In this study, we noted that macrophages in the ALI mouse model triggered by LPS had decreased levels of CPT1A level. By constructing mice harbouring a conditional depletion of macrophage CPT1A (Cre^+^ *CPT1A* ^fl/fl^), we identified a significant protective role of CPT1A in reducing lung inflammatory damage in ALI mice, as well as its anti‐inflammatory and mitochondria‐regulatory effects on bone marrow‐derived macrophages (BMDMs) challenged with LPS. Mechanistically, CPT1A could modulate metabolic alteration and polarisation of macrophage under LPS stimulation. Meanwhile, CPT1A can upregulate the expression of IL‐10/IL‐10 Receptor Alpha (IL‐10RA) by activating IL‐10 transcription factor. The introduction of exogenous IL‐10 into the Cre^+^ *CPT1A* ^fl/fl^ macrophage successfully reversed the metabolic process related to the inflammatory response triggered by LPS, revealing the crucial role of the CPT1A‐IL‐10 axis in the regulation of macrophage polarisation in the progression of ALI/ARDS.

## 2 MATERIALS AND METHODS

### 2.1 Study approval

All the experimental mice were performed in line with the guidelines established by Shanghai Committee for Accreditation of Laboratory Animal. The protocol has been officially approved by the Laboratory Animal Research Center Review Board, Tongji University, Shanghai (approval number: TJBB03721106). Pentobarbital sodium was used as an anaesthetic during the operation, and related measures were taken to reduce the discomfort of experimental animals.

### 2.2 LPS‐induced ALI mouse model

The control (C57BL/6JNju), Wild type (WT) mice, B6.129P2‐*Il10* ^tm1/Nju^ (*IL‐10* ^−/−^) mice, B6;129S‐*Cpt1a* ^tm1(flox)Smoc^ mice (Cre^−^ *CPT1A* ^fl/fl^ and Cre^+^ *CPT1A* ^fl/fl^) (6–8 weeks old, both male and female), and C57BL/6 mice (6–8 weeks old, male) were all acquired from Shanghai Model Organisms Center, Inc. (Shanghai, China). To create the conditional *Cpt1a* ^tm1(flox)Smoc^ mice, a traditional embryonic stem (ES)‐based targeting strategy was employed. The vector, linearised and transfected into SCR012ES cells using an electroporation technique, contained a 2.7 kb 5′ homology arm, a .9 kb flox region, PGK‐Neo‐polyA, a 5.1 kb 3′ homology arm and an MC1‐TK‐polyA negative selection marker. Positive ES cells were expanded and injected into the blastocysts of C57BL/6J mice to produce the first generation of Cpt1a^flox^ mice. The target mice were then generated by breeding Lyz2^cre^ mice (obtained from Shanghai Model Organisms Center) with Cpt1a^flox^ mice. The mice in the experiment were placed in a pathogen‐free environment, with a standard light/dark cycle and unrestricted intake of food and water. The animals were treated with LPS (10 mg/kg, Sigma‐Aldrich, L2630, USA) or its equivalent PBS by intranasal administration with or without simultaneous treatment with recombinant mouse IL‐10 (rIL‐10) (45 µg/kg, Biolegend, 575806, USA).^21^ Mice were sacrificed 24 h time point after LPS stimulation for subsequent analysis of inflammation and damage.

### 2.3 Bronchoalveolar lavage fluid (Balf) collection for total and differential leukocytes counting

After anaesthetisation, Balf was collected through intratracheal injection of 800 µL ice‐cold sterile PBS, followed by carefully withdraw. Balf volume from each mouse was >  70% of the injected volume and basically consistent. The Balf samples were centrifuged for 10 min at 140 × *g* at 4°C. The Balf supernatant was stored at −80°C for subsequent dissection, while the deposited cells were suspended in PBS and the total number of cells was determined by means of a haemocytometer (treated with an equal volume of 3% glacial acetic acid to lyse red blood cells). The cells in the Balf were then transferred onto glass slides using a CytoSpin (StatSpin, USA) device and stained with Wright‐Giemsa (Baso Diagnostics, China) dye. Then, using a blinded method, differential leukocytes were classified and counted in a microscope on at least 200 cells per slide.

### 2.4 Local and systemic cytokines analysis

Blood samples were collected using a syringe, .5–.8 mL of fluid is extracted via cardiac puncture and transferred to an Eppendorf Micro Test tube. After the sample has been left at room temperature for at least 1 h, the serum of mice is separated by centrifugation at 2400 × *g* for 10 min. In line with the manufacturer's guidelines, the content of cytokines in Balf supernatant and serum of experimental mice was accurately evaluated by ELISA kit. Cytokine detection covers several products of Multisciences Biotech from China, included Interferon‐γ (IFN‐γ) (EK280/3‐96), interleukin‐6 (IL‐6) (EK206/3‐96), granulocyte colony‐stimulating factor (G‐CSF) (EK269/2‐96), CXC ligand 1/keratinocyte‐derived khemokine (CXCL1/KC) (EK296/2‐96) and IL‐10 (70‐EK210/4‐96).

### 2.5 Histopathological and immunologic analysis of mouse lung

In a separate experiment, mice's left lungs from different groups, without performing bronchoalveolar lavage, were dissected and fixed in 4% paraformaldehyde. The fixed lung tissue was then bedded in paraffin and made into 5 µm slices, which were then stained with a mixture of haematoxylin and eosin (H & E staining). An image capture process (involving six randomly generated sections) was performed, followed by an assessment of the blind identity using the ALI scoring mechanism^22^ by two separate researchers already described. The degree of lung function impairment was assessed by taking into account five histological markers, including: (1) neutrophils in the alveolar space, (2) neutrophils in the interstitial space, (3) hyaline membranes, (4) proteinaceous debris filling the airspace, and (5) alveolar septal thickening. According to the conventional evaluation criteria, evaluation results of each element are zero, one or two points. These five factors are weighted according to the strength of association with ALI, while the normalisation process is performed according to the total number of fields. To assess the final damage, apply the following calculation (a continuous value number between 0 and 1): total injury score = [20 × (1) + 14 × (2) + 7 × (3) + 7 × (4) + 2 × (5)]/(number of fields × 100).

Additionally, the slides were immune‐stained with anti‐CPT1A (Abcam, ab234111, UK, 1:1000), anti‐IL‐10 (Servicebio, GB11108‐100, China, 1:500), and anti‐IL‐10RA (Abcam, ab225820, 1:100) antibodies to evaluate their distribution and expression. All images were captured using a Nikon Eclipse C1 microscope (Nikon, Japan), and then processed using ImageJ software (version 1.44p, National Institutes of Health, USA).

### 2.6 Extraction and culture of mouse BMDMs

BMDMs in the tibias and femurs of distinct mice (6–8 weeks old) were isolated following previously described methods.^23^ The tibias and femurs were cut and flushed with a 1 mL syringe. Bone marrow cells were isolated by subjecting the suspension to a centrifugal force of 220 × *g* for 5 min under room temperature. Cells were then placed in Dulbecco's modified Eagle's medium (DMEM, Gibco, USA) containing antibiotic cocktail 1% (penicillin‐streptomycin), Fetal Bovine Serum 10% (Qualified Heat Inactivated, Gibco, USA), and macrophage colony stimulating factor (M‐CSF, 20 ng/mL, PeproTech, 315‐02, USA). Such culture conditions were maintained for 7 days in order to stimulate the differentiation process of the cells. At the time of the fourth day, an equal amount of fresh complete medium (corresponding to half of the initial amount) was introduced into the cell culture dish. At the end of 7 days, the attached macrophages were harvested for further study.

### 2.7 Cell viability assay

Cell Counting Kit‐8 (Dojindo, Kumamoto, Japan) was used to evaluate the effect of different treatments on cell viability. In summary, BMDMs were seeded in 96‐well plates (the concentration is 5 × 10^4^ cells every well) and allowed to incubate overnight at 37°C. After various treatments for 12/24 h, the cells were incubated at 37°C for approximately 1 to 2 h. During this time, 10 microliters of working reagent was added to each well, in line with the manufacturer's guidelines strictly. Subsequently, the absorbance at 450 nm wavelength was quantitatively analysed with the help of iMark microplate reader (Molecular Devices, Sunnyvale, USA).

### 2.8 Quantitative real‐time PCR (qRT‐PCR)

Total BMDMs RNA was extracted by Trizol reagent (Invitrogen, USA), followed by PrimeScript™ RT Master Mix (Takara, Japan, RR036A) converts RNA to cDNA. When using the QuantStudio™ 6 Flex Real‐Time System, the qRT‐PCR was executed by the TB Green Premix Ex Taq™ (Rox) (Takara, Japan, RR420A). 2^−△△Ct^ method was used to quantitatively analyse the relative expression of mRNA. A series of specific primers produced in BioTNT (China) include *lactate dehydrogenase A (LDHA), hypoxia‐inducible factor‐1 alpha (HIF‐1α), glucose transporter (GLUT), sterol regulatory element‐binding protein (SREBP), SREBP cleavage‐activating protein (SCAP), low‐density lipoprotein receptor (LDLr), IL‐1β, IL‐6, IL‐18, tumour necrosis factor (TNF)‐α, iNOS, IL‐12, IL‐10, IL‐10RA, PBX1, PREP1, MEIS1, ARG1, YM1* and *β‐actin*. Their sequences are detailed in Table S1.

### 2.9 Measurement of mitochondrial integrity and activity

BMDM cells were placed in a 4‐well imaging chambers containing cover‐glass bottom, and they were treated with LPS and/or rIL‐10 for 24 h. Thereafter, BMDMs were washed three times with PBS, a step intended to remove the growth medium. Subsequently, the cells were incubated with MitoTracker Red FM (Invitrogen, M22425, USA) for half an hour in order to stain and label the mitochondria. The cell nuclei were stained with Hoechst (Beyotime, C1017, China) prior to confocal imaging. Mitochondrial integrity was assessed using confocal microscopy (TCS SP8, Leica, Germany).

Mitochondrial ROS‐scavenging activity was evaluated using the MitoSOX Red (Invitrogen, M36008, USA) assay. Specifically, in this experiment, BMDM cells were seed into 96‐well black plates with 5 × 10^4^ cells every well, and then expose to LPS and/or rIL‐10, respectively, for 24 h. BMDMs were then washed and incubated with 5 µM MitoSOX Red dye at 37°C in the dark for half an hour. Subsequently, a thorough washing operation was performed to wash the BMDM cells using an ice‐cold PBS solution. Subsequently, the average intensity of the fluorescence signal was measured and recorded with the aid of a SpectraMax M5/M5e fluorescent plate reader (Molecular Devices, USA) at wavelengths of 510/580 nm.

DCFH‐DA (Sigma‐Aldrich, D6883, USA) was used to quantitatively analyse the generation of ROS in cells in cooperation with the fluorescence detection device. In summary, this experiment used 96‐well black plates seeded with BMDMs at a cell density of 5×10^4^ cells per well, followed by continuous 24 h treatment with LPS and/or rIL‐10. Cells were then washed, soaked in 10 µM DCFH‐DA, and stained at 37°C for half an hour in the absence of light. After three washes with cold PBS, the content of ROS was measured at 488/525 nm wavelength using SpectraMax M5/M5e fluorescent plate reader (Molecular Devices, USA).

### 2.10 Immunoblotting assay

By immunoblotting technique, for CPT1A, dynamin‐related protein 1 (DRP1), Fission1 (FIS1), optic atrophy 1 (OPA1), mitofusin 2 (MFN2), ARG1, iNOS, NLRP3, Caspase‐1 p10, and β‐actin were analysed. The total amount of protein in different types of murine BMDMs was extracted by RIPA lysate (Beyotime, China), then the concentration was measured using the BCA protein assay kit (Beyotime, China). Firstly, protein samples were analysed by 10% SDS‐Page gel electrophoresis, and protein bands were transferred to PVDF membrane. Next, the membrane was blocked with a mixture of Tris‐buffered saline with Tween 20(TBST, BioTNT, China) and 5% skim milk. The membrane was then incubated at 4°C and conjugated to a series of primary antibodies including anti‐CPT1A (Abcam, ab234111, UK), DRP1 (Cell Signaling Technology, CST, 8570, USA), FIS1 (CST, 32525), OPA1 (CST, 80471), MFN2 (CST, 9482), NLRP3 (CST, 15101), ARG1 (CST, 93668), iNOS (CST, 13120), Caspase‐1 p10 (Abcam, ab179515), and β‐actin (CST, 3700) at a dilution ratio of 1:1000. Next, the membrane was placed in a room temperature environment together with a secondary antibody, Anti‐rabbit IgG, HRP‐linked Antibody (CST, 7074), and Anti‐mouse IgG, HRP‐linked Antibody (CST, 7076), dilution ratio 1:2000. Two hours of culture bonding were performed. After three TBST washes, the bands were visualised using a Tanon 5200 Multifunction Automated Chemiluminescence Imaging System (Tanon Biotechnology, China), and the images were analysed using the ImageJ software.

### 2.11 Seahorse metabolic analysis

According to the manufacturer's operation manual, the XFe96 analyser of Agilent Company was used to carry out the experimental study of Seahorse. BMDMs were cultured in XFe96 plates and challenged with LPS for 12 h. Real‐time alterations in the oxygen consumption rate (OCR) of BMDMs were measured following the injection of 2 µM oligomycin, 1 µM FCCP, and a combination of .5 µM rotenone and antimycin A at specified time points. For extracellular acidification rate (ECAR) measurements, where indicated, cells were sequentially injected with 10 mM glucose, 1 µM oligomycin, and 100 mM 2‐DG. To evaluate FAO, BMDMs were pretreated with either bovine serum albumin alone (BSA) or palmitate‐conjugated BSA. The ratio of the oxygen consumption rate in cells with and without exogenous palmitic acid was used to assess the level of exogenous palmitate oxidation and thus detect FAO levels.

### 2.12 Determination of citrate, acetyl CoA and ATP

The production of citrate, acetyl CoA, and ATP in BMDMs from various groups was evaluated using the Citrate Assay Kit (Abcam, ab83396, UK), PicoProbe Acetyl CoA Assay Kit (Abcam, ab87546, UK), and Luminescent ATP Detection Assay Kit (Abcam, ab113849, UK), following the manufacturer's protocols. The formation of citrate, acetyl CoA and ATP in BMDMs from different cohorts was quantitatively evaluated. The concentrations of citric acid and acetyl CoA were accurately measured at 535/587 nm wavelength using the fluorescent plate reader. ATP levels were measured in cell lysates using a luminescence‐based assay.

### 2.13 Phagocytosis experiment

The phagocytic capacity of the BMDMs was detected using pHrodo Green *E. coli* BioParticles Conjugate (Invitrogen, P35366, USA), operated in line with the manufacturer's protocols. The phagocytic capacity was determined by both fluorescence plate reader and confocal microscopy at 509/533 nm.

### 2.14 The construction of single‐cell RNA sequencing (scRNA‐seq) library and data analysis

The construction of the scRNA‐seq library (Genome Sequence Archive with accession ID CRA008837) using the 10× Genomics platform was described in detail previously.^21^ Briefly, the lung tissue is discreetly cut into small pieces and subjected to a decomposition process in order to obtain a suspension consisting of individual cells, which is then filtered by means of a 100‐µm cell strainer. Processing of the fresh cell suspension was performed in strict accordance with the immediate instructions of the manufacturer of the 10× Chromium 3′ v3 kit (10× Genomics, Pleasanton, CA). On the NovaSeq 6000 platform (Illumina, Inc., San Diego, CA), the library was construction, as well as the sequence analysis was performed. The initial sequence read data was converted to fastq files using Illumina bcl2fastq2 Conversion Software v2.20 (available at https://support.illumina.com/downloads/bcl2fastq‐conversion‐software‐v2‐20.html). Utilise FastQC software v0.11.9 (available at https://www.bioinformatics.babraham.ac.uk/projects/fastqc/) to rigorously evaluate the quality of sequence data. Standard cell ranger pipelines were used for sequence processing and alignment to the GRcm39 genome with default parameters (https://support.10xgenomics.com/single‐cell‐gene‐expression/software/pipelines/latest/). Cell samples from WT control mice and WT LPS‐treated mice were finally analysed, with 1129 cells in the control group and 4478 cells in the LPS‐treated group. By applying a non‐linear dimensionality reduction technique and a graphical model clustering method, a series of key cluster divisions are set according to the prototype marker, included are epithelial cells (identified by *Epcam*), endothelial cells (identified by *Pecam1*), fibroblasts (identified by *Col1a2*), neutrophils (identified by *Ly6g*), monocytes–macrophages (identified by *Fcgr1* and *Mrc1*), T‐lymphocytes (characterised by *Cd3e*), B lymphocytes (marked by *Cd19*), dendritic cells (indicated by *Cd83*) and natural killer cells (marked by *Klrb1c*) (Figure S1a and b). By applying the likelihood‐ratio test of single‐cell gene expression, genes with significant differences in expression can be identified. This approach is similar to the one implemented by Seurat's v6 FindAllMarkers function.

### 2.15 RNA‐sequence library preparation and data processing

A total of 3 × 10^6^ BMDM cells per well were seeded in the 6 cm dish, and then the cells were exposed to LPS for 12 h. Cell samples were then collected and Trizol reagent (Invitrogen, Carlsbad, CA, USA) was applied in line with the manufacturer's manual to obtain total RNA. RNA samples were identified by spectrophotometric analysis, followed by detailed quality assessment using the Agilent 2100 Bioanalyzer (Agilent Technologies). The RNA integrity of all test samples remained good, with scores generally exceeding 7.0. A sequencing library is construct by a kit named TruSeq Stranded mRNA LT Sample Prep Kit (Illumina, CA, USA). The library was subsequently sequenced on the Illumina Novaseq 6000 platform (Macrogen, Seoul, South Korea). The platform uses a 2 × 150bp paired‐end read sequence.

Fastp software (https://github.com/OpenGene/fastp) tool removes reads that contain linker contamination, low‐quality bases and indeterminate bases. The quality of the sequence was checked using the fastp tool. HISAT2 (https://ccb.jhu.edu/software/hisat2) was aligned to map reads to the Mouse Genome Assembly GRCm39 reference genome. The mapped reads of each sample were assembled by StringTie (https://ccb.jhu.edu/software/stringtie). The official website of the StringTie should operate with its default configuration. Subsequently, all transcriptomes from all samples were merged to reconstruct a comprehensive transcriptome by gffcompare (https://github.com/gpertea/gffcompare). Table attainment was assessed for all transcripts in units of FPKM (i.e., the number of complete exon fragments/mapped reads in millions × exon length in kB). Significantly differentially expressed mRNAs were detected with a fold change of more than 2 or less than .5 and *p*‐value < .05 using edgeR (https://bioconductor.org/packages/release/bioc/html/edgeR.html). Gene Ontology (GO) classification and Kyoto Encyclopedia of Genes and Genomes (KEGG) pathway of differentially expressed mRNAs were interpreted.

### 2.16 Statistical analysis

In addition to scRNA‐seq data, data are shown as mean ± standard error (± SEM) of at least three independent experiments or biological samples. Additionally, by means of GraphPad Prism software (version 8.0.1, GraphPad Software, Inc., San Diego, CA), the data obtained from the experiment were explored by performing a two‐tailed unpaired Student's *t*‐test, one‐way ANOVA followed by Bonferroni's post hoc test. The Shapiro–Wilk test is applied to assess the normality of the samples. *p* Value less than .05 was considered statistically significant. Detailed statistical values are provided in the figure legends.

## 3 RESULTS

### 3.1 Macrophage CPT1A deficiency contributes to more severe lung injury upon LPS challenge

Our previous scRNA‐seq data (Genome Sequence Archive with accession ID CRA008837) showed that *CPT1A* mainly expressed in macrophages and fibroblasts and was reduced by LPS challenge (Figure S1a–d). Meanwhile, we constructed a classical ALI mouse model via LPS inhalation (10 mg/kg) and observed reduced CPT1A expression in lung macrophages of ALI mice compared to the control (Figure S1e), which indicated a potential relationship between decreased CPT1A expression in macrophage upon LPS exposure and ALI pathogenesis.

**FIGURE 1:**
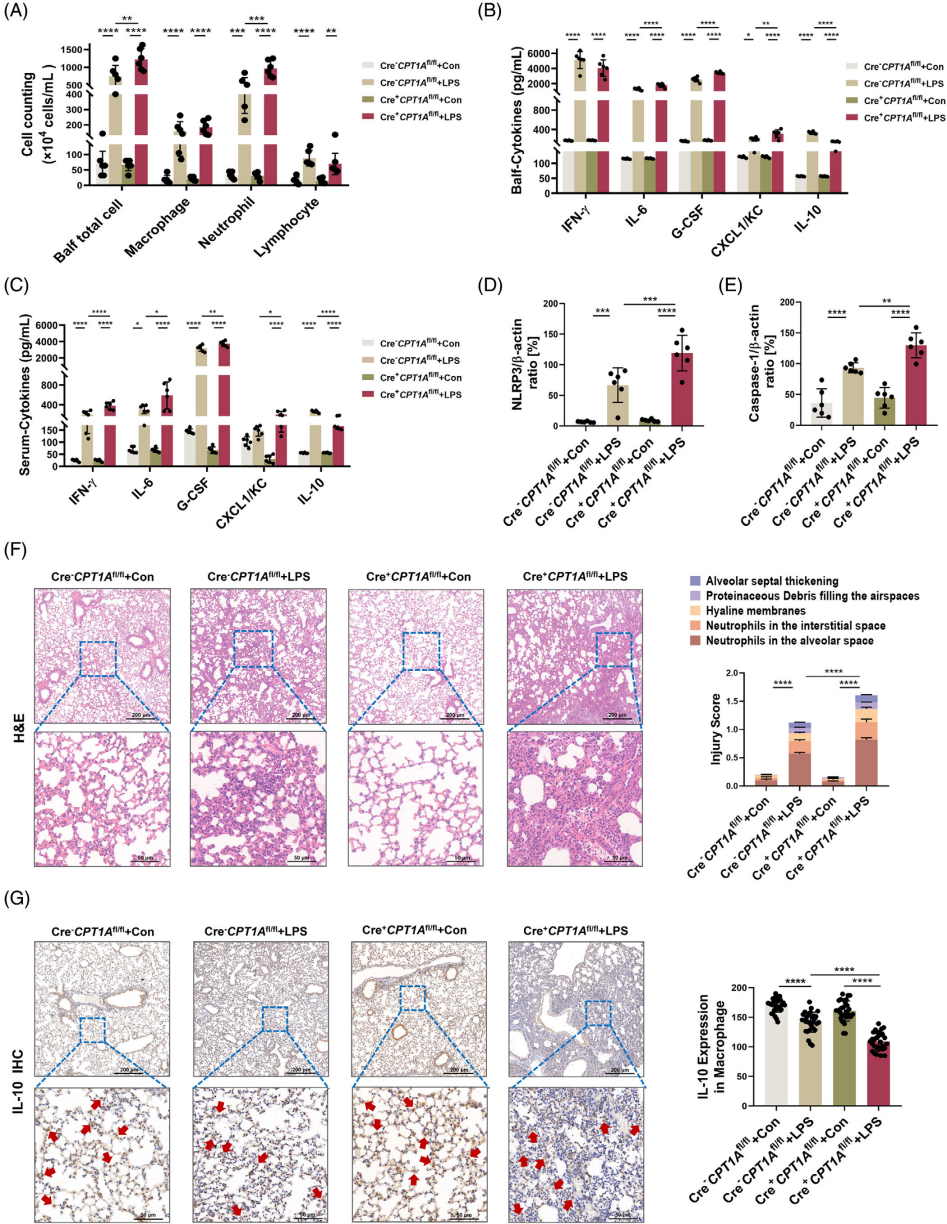
CPT1A deficiency in macrophage contributes to more severe lung injury in the ALI mouse model. (**a)** Cre^−^ *CPT1A* ^fl/fl^ and Cre^+^ *CPT1A* ^fl/fl^ mice were administrated nasally with PBS or LPS (10 mg/kg). After 24 h, mice were euthanised and Balf was collected and estimated for total cell number, macrophage, neutrophil and lymphocyte numbers. *n* = 6 biologically independent samples. Balf total cell, **p = .0044, ****p < .0001. Macrophage, ****p < .0001. Neutrophil, ***p = .0002, ****p < .0001. Lymphocyte, **p = .002, ****p < .0001. (**b, c)** Secretion of cytokines IFN‐γ, IL‐6, G‐CSF, CXCL1/KC and IL‐10 in Balf and serum was assessed by ELISA kits. *n* = 6 biologically independent samples. (**b**) *p = .0212, **p = .0017, ****p < .0001. (**c**) *p = .0368 (Cre^−^ *CPT1A* ^fl/fl^+Con group vs. Cre^−^ *CPT1A* ^fl/fl^+LPS group), *p = .0116 (Cre^−^ *CPT1A* ^fl/fl^+LPS group vs. Cre^+^ *CPT1A* ^fl/fl^+LPS group), **p = .0046, *p = .0448, ****p < .0001. (**d, e)** Quantitative analysis of immunoblotting assay identifying the activation of NLRP3/Caspase‐1 pathway for lung inflammation evaluation. *n* = 6 biologically independent samples. (**d**) ***p = .0002 (Cre^−^ *CPT1A* ^fl/fl^+Con group vs. Cre^−^ *CPT1A* ^fl/fl^+LPS group), ***p = .0009 (Cre^−^ *CPT1A* ^fl/fl^+LPS group vs. Cre^+^ *CPT1A* ^fl/fl^+LPS group), ****p < .0001. (**e**) **p = .0086, ****p < .0001. (**f)** Representative pictures of H&E‐stained lung tissue sections from each group. Scale bars in the upper panel, 200 µm; scale bars in the lower panel, 50 µm. Lung injury score was determined by 5 pathophysiological features. *n* = 6 biologically independent samples. ****p < .0001. (**g)** Immunohistochemical staining and quantitative analysis of IL‐10 in Cre^−^ *CPT1A* ^fl/fl^ and Cre^+^ *CPT1A* ^fl/fl^ mice. Red arrowheads indicate IL‐10‐expressed macrophage within lungs. *n* = 30 each group from 6 biologically independent samples. ****p < .0001. Scale bars in the upper panel, 200 µm; scale bars in the lower panel, 50 µm. Data are presented as mean  ±  SEM and analysed with a 95% confidence interval. *p* Values were calculated using one‐way ANOVA followed by Bonferroni's post hoc test.

To prove this, we generated mice harbouring a conditional depletion of CPT1A in macrophages (Cre^+^ *CPT1A* ^fl/fl^). By use of the ALI mouse model, control (Cre^−^ *CPT1A* ^fl/fl^) and Cre^+^ *CPT1A* ^fl/fl^ mice were injected with LPS or PBS. Their local and systemic inflammatory responses were then measured and analysed 24 h after treatment. As shown in Figure 1a, Balf inflammatory cell infiltration was dramatically elevated by LPS challenge, especially neutrophils, and was further exacerbated by CPT1A deficiency in macrophages. IFN‐γ, IL‐6, G‐CSF and CXCL1/KC in Balf and serum were elevated rapidly after LPS stimulation, while CPT1A depletion in macrophages intensified the increase at 24 h except for IFN‐γ in Balf (Figure 1b and c). We also observed elevated IL‐10 level in both Balf and serum after LPS challenge. Interestingly, the level of IL‐10 was much lower in Cre^+^ *CPT1A* ^fl/fl^ mice compared with the Cre^−^ *CPT1A* ^fl/fl^ ones (Figure 1b and c).

To further estimate the role of macrophage CPT1A expression in LPS‐triggered inflammatory lung injuries, we applied whole lung lysates for immunoblotting detection and lung sections for histological staining. In Figures S2a and 1d and e, we observed that the NLRP3/Caspase‐1 signalling pathway associated with inflammatory response in ALI mouse lung was promoted when CPT1A gene was absent in macrophages. Lung structure can be evaluated from five different perspectives: observing the alveolar neutrophils, assessing the interstitial neutrophils, detecting the hyaline membranes, analysing the proteinaceous debris and measuring the septal thickening. We observed no obvious pathological change when depleting macrophage CPT1A under physiological condition; however, exacerbated pulmonary damage in Cre^+^ *CPT1A* ^fl/fl^ mice could be identified compared with the Cre^−^ *CPT1A* ^fl/fl^ ones at 24 h after LPS stimulation (Figure 1f).

Next, we evaluated the effect of CPT1A on IL‐10 expression on tissue scales by staining mouse lung sections with IL‐10 and its receptor, IL‐10RA. After LPS inhalation, infiltrated macrophages lack of CPT1A expressed lower levels of IL‐10 (Figure 1g), as well as IL‐10RA (Figure S2b) compared with Cre^−^ *CPT1A* ^fl/fl^ mice. In a nutshell, the expression of CPT1A gene in BMDMs shows a crucial role in the progression of ALI induced by LPS, which can affect the regulation of IL‐10 and its receptor.

### 3.2 CPT1A regulates aberrant inflammation and mitochondrial stability in macrophages upon LPS challenge

**FIGURE 2:**
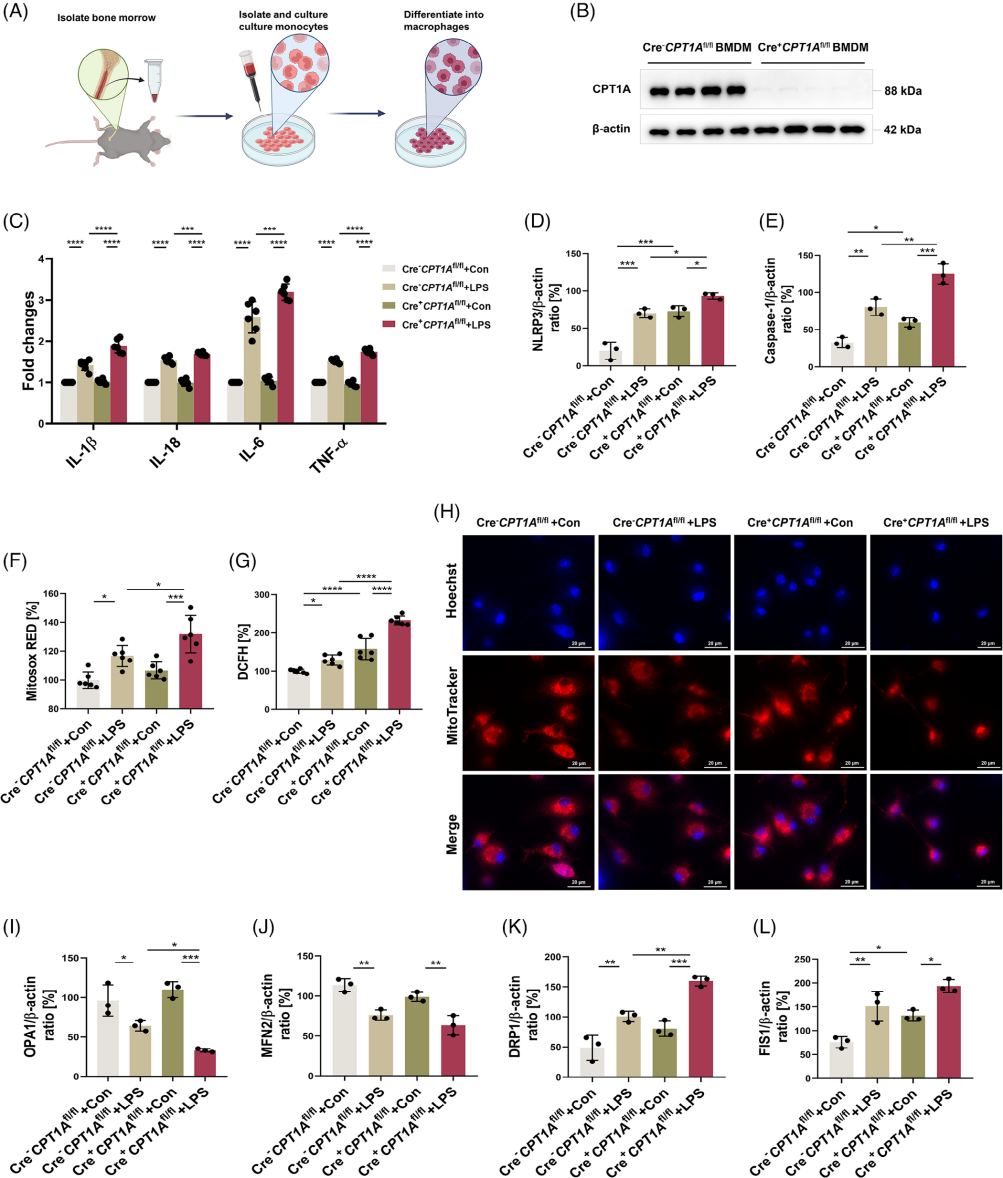
CPT1A regulates aberrant inflammation and mitochondrial stability in macrophages upon LPS challenge. **(a)** Schematic diagram of the extraction, separation and cultivation of BMDMs in vitro. Some figure elements were created with BioRender.com. (**b)** Macrophage CPT1A depletion was verified by immunoblotting in BMDMs from Cre^−^ *CPT1A* ^fl/fl^ and Cre^+^ *CPT1A* ^fl/fl^ mice. *n* = 4 biologically independent samples. (**c)** After LPS stimulation (100 ng/mL) for 6 h, the induction of cytokines IL‐1β, IL‐18, IL‐6 and TNF‐α mRNA expression in each group was analysed by qRT‐PCR. Data are expressed as fold change. *n* = 6 biologically independent samples. ***p = .0007, ***p = .0004, ****p < .0001. (**d, e)** After LPS (100 ng/mL) treatment for 24 h, quantitative immunoblotting analysis indicating the effect of CPT1A expression on NLRP3/Caspase‐1 p10 level for inflammasome activation in BMDMs. *n* = 3 biologically independent samples. (**d**) ***p = .0002 (Cre^−^ *CPT1A* ^fl/fl^+Con group vs. Cre^−^ *CPT1A* ^fl/fl^+LPS group), ***p = .0001 (Cre^−^ *CPT1A* ^fl/fl^+Con group vs. Cre^+^ *CPT1A* ^fl/fl^+Con group), *p = .0451 (Cre^+^ *CPT1A* ^fl/fl^+Con group vs. Cre^+^ *CPT1A* ^fl/fl^+LPS group), *p = .0243 (Cre^−^ *CPT1A* ^fl/fl^+LPS group vs. Cre^+^ *CPT1A* ^fl/fl^+LPS group). (**e**) **p = .0015 (Cre^−^ *CPT1A* ^fl/fl^+Con group vs. Cre^−^ *CPT1A* ^fl/fl^+LPS group), *p = .0411, ***p = .0002, **p = .0023 (Cre^−^ *CPT1A* ^fl/fl^+LPS group vs. Cre^+^ *CPT1A* ^fl/fl^+LPS group). (**f, g)** Microplate reader assay showing the effect of CPT1A on the production of mitochondrial (**f**) and intracellular ROS (**g**) induced by LPS (100 ng/mL) in BMDMs. *n* = 6 biologically independent samples. (**f**) *p = .0114 (Cre^−^ *CPT1A* ^fl/fl^+Con group vs. Cre^−^ *CPT1A* ^fl/fl^+LPS group), ***p = .0002, *p = .0243. (**g**) *p = .0268, ****p < .0001. (**h)** Confocal microscopy images showing the mitochondrial mass of BMDMs from each group using MitoTracker probe. Scale bars, 20 µm. (**i–l)** Quantitative immunoblotting analysis indicating the effect of CPT1A on the expression of OPA1, MFN2, DRP1 and FIS1 for mitochondrial dynamics in BMDMs. *n* = 6 biologically independent samples. (**i**) *p = .013 (Cre^−^ *CPT1A* ^fl/fl^+Con group vs. Cre^−^ *CPT1A* ^fl/fl^+LPS group), ***p = .0002 (Cre^+^ *CPT1A* ^fl/fl^+Con group vs. Cre^+^ *CPT1A* ^fl/fl^+LPS group), *p = .0397 (Cre^−^ *CPT1A* ^fl/fl^+LPS group vs. Cre^+^ *CPT1A* ^fl/fl^+LPS group). (**j**) **p = .0026 (Cre^−^ *CPT1A* ^fl/fl^+Con group vs. Cre^−^ *CPT1A* ^fl/fl^+LPS group), **p = .0035 (Cre^+^ *CPT1A* ^fl/fl^+Con group vs. Cre^+^ *CPT1A* ^fl/fl^+LPS group). (**k**) **p = .0063 (Cre^−^ *CPT1A* ^fl/fl^+Con group vs. Cre^−^ *CPT1A* ^fl/fl^+LPS group), **p = .0028 (Cre^−^ *CPT1A* ^fl/fl^+LPS group vs. Cre^+^ *CPT1A* ^fl/fl^+LPS group), ***p = .0004. (**l**) *p = .027 (Cre^−^ *CPT1A* ^fl/fl^+Con group vs. Cre^+^ *CPT1A* ^fl/fl^+Con group), *p = .0154 (Cre^+^ *CPT1A* ^fl/fl^+Con group vs. Cre^+^ *CPT1A* ^fl/fl^+LPS group), **p = .0049. Data are presented as mean  ±  SEM and analysed with a 95% confidence interval.  *p* Values were calculated using one‐way ANOVA followed by Bonferroni's post hoc test.

We then investigated the effects of CPT1A on inflammatory response and metabolic patterns of macrophage ex vivo by isolating and culturing mouse bone marrow derived macrophages (BMDMs) (Figure 2a). Macrophage CPT1A depletion was verified by immunoblotting that BMDMs isolated from Cre^+^ *CPT1A* ^fl/fl^ mice showed a complete absence of CPT1A expression (Figure 2b). BMDMs from Cre^−^ *CPT1A* ^fl/fl^ and Cre^+^ *CPT1A* ^fl/fl^ mice were stimulated with LPS and subjected to RNA‐seq. Heatmaps of all differential gene expression results were presented in Figure S3a. A total of 2357 differentially expressed genes (DEGs) were identified between Cre^−^ *CPT1A* ^fl/fl^ and Cre^+^ *CPT1A* ^fl/fl^ macrophages in the control group, and in the experimental group, the number was 1069 (Figure S3b). The most highly DEGs in Cre^−^ *CPT1A* ^fl/fl^ and Cre^+^ *CPT1A* ^fl/fl^ BMDMs showed that CPT1A depletion had a significant impact on cellular metabolism (*Fabp5*, *Ldhb, Ldha, Acsbg1* and *Acadvl*) and proinflammatory cytokine production (*Nlrp3*, *Il1b, Il33*), especially under LPS stimulation (Figure S3c). In terms of consistency, it was observed that the enrichment analysis of GO presented a similar pattern with the pathway analysis of KEGG (Figure S3d and e). By use of qRT‐PCR, we determined that in the absence of CPT1A, LPS administration induced more IL‐1β, IL‐18, IL‐6 and tumour necrosis factor (TNF)‐α, cytokines that promote inflammation production (Figure 2c). In addition, we also investigated the mechanism of NLRP3 inflammasome activation, which is responsible for the secretion of IL‐1β and IL‐18.^24^ The results showed that loss of CPT1A enhanced LPS‐induced the NLRP3/Caspase‐1 signalling pathway activation in BMDMs (Figures S4a and 2d and e).

CPT1A mainly localised in mitochondrial membrane and has been reported to maintain mitochondrial homeostasis and attenuate excessive inflammatory response in renal tubular epithelial cells under profibrotic stimuli.^25^ To answer the question of whether macrophage CPT1A expression prevented mitochondrial dysfunction, we examined ROS generation after LPS stimulation and discovered that LPS‐induced mitochondrial and intracellular ROS level elevated in macrophages with or without CPT1A, and Cre^+^ *CPT1A* ^fl/fl^ BMDMs exhibited even higher ROS level compared to Cre^−^ *CPT1A* ^fl/fl^ cells using MitoSOX Red probe and DCFH‐DA fluorescent probe under stimulus, respectively (Figure 2f and g), demonstrating the potential mitochondrial ROS‐scavenging activity of CPT1A in macrophages. Thereafter, we stained and imaged the active mitochondria in the cell using MitoTracker fluorescent dye, and observed that a decrease in fluorescence brightness indicates signs of mitochondrial damage. As revealed in Figure 2h, the fluorescence intensity of MitoTracker was decreased upon LPS challenge, which could be further reduced by CPT1A depletion. As a dynamic organelle, the maintenance of the mitochondrial homeostasis highly dependents on its fission and fusion process.^26^ It has been shown that once the balance between fission and fusion in mitochondria is disturbed, its oxidation is affected.^27^ To explore the potential role of CPT1A on macrophage mitochondrial dynamics, we analysed the expression levels of OPA1 and MFN2, as well as the fission proteins DRP1 and FIS1. Results showed that LPS treatment dramatically reduced OPA1 and MFN2 expression, while increased DRP1 and FIS1 expression, which indicates abnormal mitochondrial dynamics (Figures S4b and 2i–l). Besides, CPT1A depletion intensified the decrease of OPA1 and elevation of DRP1 in BMDMs (Figures S4b and 2i and k). The findings suggest that CPT1A expression participated in maintaining mitochondrial homeostasis, thereby protecting macrophages against excess inflammation.

Since genetic inactivation of CPT1A can disrupt mitochondrial homeostasis and induce inflammation, we asked whether pharmacological CPT1A modulation could augment bioenergetic and inflammatory reaction under LPS stimulation. To test this, we applied the well‐established CPT1A activator L‐carnitine (Lca), as well as its inhibitor etomoxir (Eto),^15^ and proved their independence of the cytotoxicity in BMDMs (Figure S5a and b). Compared with LPS group, L‐carnitine significantly increased the level of CPT1A expression, which could be restrained by etomoxir pretreatment (Figure S5c). Lca can significantly down‐regulate the levels of inflammatory mediators IL‐1β and TNF‐α, and effectively inhibit the activation of NLRP3/Caspase‐1 signalling pathway, which has been clearly verified in the data. At the same time, etomoxir showed a trend opposite to our observations (Figure S5d–f). Additionally, Lca prevented abnormal mitochondrial dynamics by elevating fusion proteins (OPA1, MFN2) and reducing the fission protein DRP1; conversely, CPT1A inhibition by etomoxir decreased fusion proteins MFN2 and increased mitochondrial fission protein FIS1 (Figure S5g), which further demonstrated the importance of CPT1A in regulating the abnormal inflammatory response and mitochondrial stability of macrophages stimulated by LPS.

### 3.3 CPT1A modulates metabolic reprogramming from glycolysis to FAO in LPS‐stimulated macrophages

**FIGURE 3:**
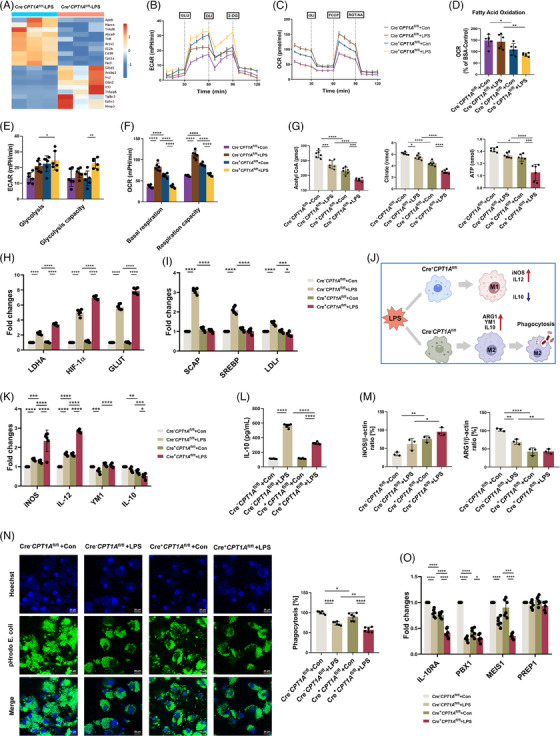
CPT1A modulates metabolic reprogramming from glycolysis to FAO, polarisation and IL‐10 production in LPS‐stimulated macrophages. **(a)** Heat map of DEGs in Cre^−^ *CPT1A* ^fl/fl^ and Cre^+^ *CPT1A* ^fl/fl^ BMDMs after 12 h stimulation with LPS (100 ng/mL). (**b, c)** ECAR and OCR of BMDMs from Cre^−^ *CPT1A* ^fl/fl^ and Cre^+^ *CPT1A* ^fl/fl^ mice were measured with a Seahorse XFe96 analyser. Where indicated, cells were injected with 10 mM glucose (GLU), 1 µM oligomycin (OLI) and 100 mM 2‐DG sequentially for ECAR (**b**). For OCR, BMDMs were pretreated with 170 µM palmitate‐BSA FAO substrate or 40 µM CPT1A inhibitor Etomoxir, followed by injection of 2 µM OLI, 1 µM FCCP, and a combination of .5 µM rotenone and antimycin A (AA) at the time points indicated (**c**). *n* = 6 biologically independent samples at each data point. (**d–f)** The FAO level (**d**), glycolysis level and glycolysis capacity (**e**), and basal respiration and respiration capacity (**f**) of BMDMs from Cre^−^ *CPT1A* ^fl/fl^ and Cre^+^ *CPT1A* ^fl/fl^ mice with or without LPS stimulation. *n* = 6 biologically independent samples. (**d**) *p = .0312, **p = .0019. **(e**) *p = .0329, **p = .0022. (**f**) ****p < .0001. (**g)** After 12 h treatment with LPS (100 ng/mL) in BMDMs, levels of intracellular FAO metabolites, acetyl CoA and citrate, as well as ATP were measured. *n* = 6 biologically independent samples. Acetyl CoA, ***p = .0001 (Cre^−^ *CPT1A* ^fl/fl^+Con group vs. Cre^−^ *CPT1A* ^fl/fl^+LPS group), ***p = .0006 (Cre^+^ *CPT1A* ^fl/fl^+Con group vs. Cre^+^ *CPT1A* ^fl/fl^+LPS group), ****p < .0001. Citrate, *p = .0241, ****p < .0001. ATP, *p = .0449, ***p = .0001, ****p < .0001. (**h, i)** Induction of LDHA, HIF‐1α, GLUT, SCAP, SREBP and LDLr mRNA expression in Cre^−^ *CPT1A* ^fl/fl^ or Cre^+^ *CPT1A* ^fl/fl^ BMDMs was estimated by qRT‐PCR. Data are expressed as fold change. *n* = 6 biologically independent samples. (**h**) ****p < .0001. (**i**) *p = .0147, ***p = .0002, ****p < .0001. (**j**) Diagram of the modulatory effects of CPT1A on macrophage activation and polarisation upon LPS stimulation. Some figure elements were created with BioRender.com. (**k)** Induction of iNOS, IL‐12, YM1 and IL‐10 mRNA expression in Cre^−^ *CPT1A* ^fl/fl^ or Cre^+^ *CPT1A* ^fl/fl^ BMDMs was analysed via qRT‐PCR. Data are expressed as fold change. *n* = 6 biologically independent samples. iNOS, ***p = .0002, ****p < .0001. IL‐12, ****p < .0001. YM1, ***p = .0002, ****p < .0001. IL‐10, **p = .0012, ***p = .0005, *p = 0.0443. (**l)** The secretion level of IL‐10 by BMDMs was examined by ELISA assay 24 h after LPS stimulation (100 ng/mL). *n* = 6 biologically independent samples. ****p < .0001. (**m)** Quantitative immunoblotting analysis indicating the effect of CPT1A on ARG1 and iNOS expression for macrophage polarisation in BMDMs. *n* = 3 biologically independent samples. iNOS, **p = .007, *p = .021. ARG1, **p = .0024 (Cre^−^ *CPT1A* ^fl/fl^+Con group vs. Cre^−^ *CPT1A* ^fl/fl^+LPS group), **p = .0082 (Cre^−^ *CPT1A* ^fl/fl^+LPS group vs. Cre^+^ *CPT1A* ^fl/fl^ +LPS group), ****p < .0001. (**n)** The phagocytic capacity of BMDMs from Cre^−^ *CPT1A* ^fl/fl^ or Cre^+^ *CPT1A* ^fl/fl^ mice was evaluated by use of the pHrod Green *E. coli* BioParticles through both confocal microscopy (showed in the left panel; scale bars, 20 µm) and fluorescence plate reader (showed in the right panel). *n* = 6 biologically independent samples. *p = .045, **p = .0013, ****p < .0001. (**o)** The levels of IL‐10RA, PBX1, MEIS1 and PREP1 mRNA expression in BMDMs were analysed by qRT‐PCR. Data are expressed as fold change. *n* = 6 biologically independent samples. IL‐10RA, ****p < .0001. PBX1, *p = .0196, ****p < .0001. MEIS1, ***p = .0004, ****p < .0001. Data are presented as mean  ±  SEM and analysed with a 95% confidence interval. *p* Values were calculated using one‐way ANOVA followed by Bonferroni's post hoc test.

The heatmap of DEGs showed that many FAO‐associated genes were down‐regulated by CPT1A depletion in LPS‐stimulated BMDMs compared with the Cre^−^ *CPT1A* ^fl/fl^ group (Figure 3a). To further assess the effect of CPT1A expression on metabolic plasticity upon activation, we examined ECAR and OCR in BMDM isolated from Cre^−^ *CPT1A* ^fl/fl^ and Cre^+^ *CPT1A* ^fl/fl^ mice. We determined glycolytic function by oligomycin‐induced OXPHOS blockage and discovered that absence of CPT1A promoted basal glycolytic function in the presence of LPS (Figure 3b and e). Simultaneously, we found that palmitate‐induced OCR was significantly lower in BMDMs isolated from Cre^+^ *CPT1A* ^fl/fl^ compared to Cre^−^ *CPT1A* ^fl/fl^ mice under baseline situation, suggesting a relatively low level of fatty acid metabolism. After LPS stimulation, FAO level dropped in both groups, and the decline was more significant in Cre^+^ *CPT1A* ^fl/fl^ group (Figure 3d). Compared with Cre^−^ *CPT1A* ^fl/fl^, Cre^+^ *CPT1A* ^fl/fl^ BMDMs showed a lower‐level of baseline oxygen consumption, and OCR, basal respiration, and respiration capacity elevation upon LPS challenge (Figure 3c and f). These metabolic alterations may be due to the glycolysis‐related oxygen consumption. Besides, levels of acetyl CoA, citrate and ATP in Cre^+^ *CPT1A* ^fl/fl^ macrophages was decreased compared to Cre^−^ *CPT1A* ^fl/fl^ ones both in the absence and presence of LPS, indicating a low activity of FAO‐OXPHOS (Figure 3g).

In addition, we estimated metabolic alterations of macrophages with or without CPT1A expression upon LPS challenge via key regulators measurement. We found that under LPS challenge, there was an upregulation trend in mRNA expression of LDHA, GLUT and HIF‐1α^28^, ^29^ in macrophages, and the elevation is more obvious under CPT1A knockout condition (Figure 3h). In contrast, while LPS activation enhanced the expression levels of LDLr, SREBP, and SCAP^9^, ^30^, ^31^ in Cre^−^ *CPT1A* ^fl/fl^ ones, these gene expression were obvious repressed in Cre^+^ *CPT1A* ^fl/fl^ BMDMs under the same stimulation (Figure 3i). Notably, these alterations could not be attributed to the cellular cytotoxicity induced by CPT1A defect or LPS administration (Figure S6). Together, the above results suggest that defect of FAO function in CPT1A‐absent macrophages may lead to the active mobilisation of glycolysis, and CPT1A plays a pivotal role in maintaining stable cellular energy metabolism in macrophages upon LPS challenge.

### 3.4 CPT1A shifts macrophages polarisation and modulates IL‐10 production in the ALI model

As mentioned above, FAO is linked to the M2 state of macrophage,^32^ so we speculated that CPT1A, as the major rate‐limiting enzyme of FAO, may affect the polarisation of macrophage under LPS stimulation (Figure 3j). As shown in Figure 3k, stimulation with LPS significantly increased the M1 marker proteins iNOS and IL‐12 expression, which could be further elevated by CPT1A deficiency. Conversely, M2 markers YM1 and IL‐10 gene expression was decreased in Cre^+^ *CPT1A* ^fl/fl^ BMDMs compared to the Cre^−^ *CPT1A* ^fl/fl^ ones upon LPS challenge (Figure 3k). At protein expression level, macrophage CPT1A defect enhanced M1 marker iNOS while reduced M2 marker ARG1 regardless of whether they were activated by LPS (Figures 3m and S7a). M2 macrophages display significant anti‐inflammatory properties compared to proinflammatory M1 macrophages and play a significant part in the phagocytic function.^33^ Herein, by use of the pHrod Green *E. coli* BioParticles, we observed lower phagocytosis activity of Cre^+^ *CPT1A* ^fl/fl^ BMDMs than Cre^−^ *CPT1A* ^fl/fl^ ones upon LPS administration (Figure 3n).

As we have observed a potential regulation effect of IL‐10/IL‐10RA expression by CPT1A on tissue scales, we then carried out in vivo studies which showed that both airway and peripheral IL‐10 secretion were suppressed by the depletion of macrophage CPT1A (Figure 1). Consistently, CPT1A knockout not only inhibited the mRNA level and secretion of IL‐10 (Figure 3k and l), but also reduced the expression of its receptor IL‐10RA in LPS‐stimulated BMDMs (Figure 3o), which indicates a potential autocrine effect of IL‐10 in macrophages. Additionally, CPT1A depletion also decreased the expression of PBX1, MEIS1, but not PREP1 upon LPS stimulation (Figure 3o), which could mediate transcriptional activation of IL‐10 during phagocytosis.^18^ Overall, the data provide evidence that CPT1A was able to drive the macrophages polarisation between M1/M2 phenotype and promote IL‐10 production, which might modulate the excess inflammatory response during ALI.

### 3.5 IL‐10 generation is required for the anti‐inflammatory activity of macrophages in the ALI model

**FIGURE 4:**
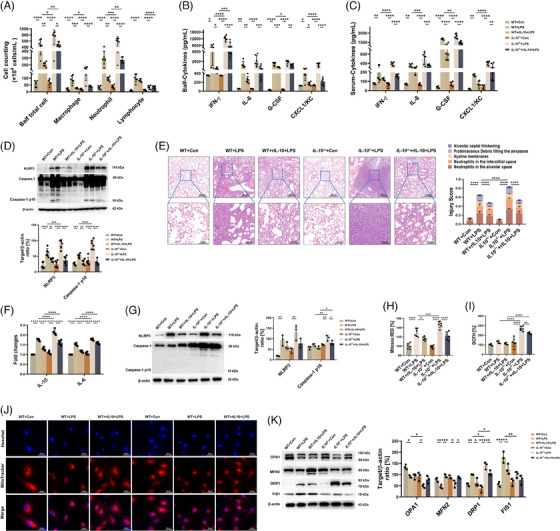
IL‐10 is essential in the restoration of ALI models, which could protect the anti‐inflammatory activities of macrophages from the mitochondrial dysfunction under LPS challenge. (**a)** WT and *IL‐10^−/−^* mice were administrated nasally with PBS or LPS (10 mg/kg) with or without rIL‐10 treatment (45 µg/kg). After 24 h, mice were euthanised and Balf was collected and estimated for total cell number, macrophage, neutrophil, and lymphocyte numbers. *n* = 6 biologically independent samples. Balf total cell, *p = .014 (WT+LPS group vs. *IL‐10* ^−/−^+LPS group), *p = .0114 (*IL‐10* ^−/−^+LPS group vs. *IL‐10* ^−/−^+rIL‐10+LPS group), **p = .0014 (WT+LPS group vs. WT+rIL‐10+LPS group), **p = .0018 (WT+rIL‐10+LPS group vs. *IL‐10* ^−/−^+rIL‐10+LPS group), ****p < .0001. Macrophage, *p = .0251, ***p = .0002, ****p < .0001. Neutrophil, **p = .0011 (WT+LPS group vs. WT+rIL‐10+LPS group), **p = .0079 (WT+rIL‐10+LPS group vs. *IL‐10* ^−/−^+rIL‐10+LPS group), ***p = .0001, ****p < .0001. Lymphocyte, ****p < .0001. (**b, c)** Production of cytokines IFN‐γ, IL‐6, G‐CSF and CXCL1/KC in Balf and serum was assessed via ELISA kits. *n* = 6 biologically independent samples. **(b)** IFN‐γ, *p = .0216 (WT+Con group vs. WT+LPS group), *p = .0333 (WT+LPS group vs. WT+rIL‐10+LPS group), ***p = .0004, ****p < .0001. IL‐6, **p = .0011 (WT+Con group vs. WT+LPS group), **p = .0064, ***p = .0002 (*IL‐10* ^−/−^+LPS group vs. *IL‐10* ^−/−^+rIL‐10+LPS group), ****p < .0001. G‐CSF, *p = .0445, **p = .0012, ***p = .0002, ****p < .0001. CXCL1/KC, *p = .0127 (WT+Con group vs. WT+LPS group), *p = .0447 (WT+LPS group vs. WT+rIL‐10+LPS group), ****p < .0001. (**c)** IFN‐γ, **p = .0039, ****p < .0001. IL‐6, **p = .0028, ***p = .0005 (*IL‐10* ^−/−^+LPS group vs. *IL‐10* ^−/−^+rIL‐10+LPS group), ***p = .0002 (WT+LPS group vs. *IL‐10* ^−/−^+LPS group), ****p < .0001. G‐CSF, **p = .0088, ****p < .0001. CXCL1/KC, **p = .0034 (WT+Con group vs. WT+LPS group), **p = .0013 (*IL‐10* ^−/−^+LPS group vs. *IL‐10* ^−/−^+rIL‐10+LPS group), ****p < .0001. (**d)** Immunoblotting and its quantitative analysis indicating the effect of IL‐10 on the NLRP3/Caspase‐1 activation for lung inflammation. *n* = 6 biologically independent samples. NLRP3, **p = .0099, ***p = .0009, ****p < .0001. Caspase‐1 p10, ***p = .0003, **p = .0042, ****p < .0001. (**e)** Representative images of H&E‐stained lung tissue sections of WT or *IL‐10* ^−/−^ mice after LPS stimulation for 24 h with or without rIL‐10 treatment. Scale bars in the upper panel, 200 µm; scale bars in the lower panel, 50 µm. Lung damage was determined by 5 pathophysiological features to obtain the total injury score. *n* = 6 biologically independent samples. ****p < .0001. (**f)** After LPS stimulation (100 ng/mL) for 6 h with or without rIL‐10 pretreatment, the mRNA expressions of cytokines IL‐1β, and IL‐6 were examined by qRT‐PCR. Data are expressed as fold change. *n* = 6 biologically independent samples. ****p < .0001. (**g)** Immunoblotting and its quantitative analysis showing the effect of IL‐10 on the NLRP3/Caspase‐1 activation for macrophage inflammation after 24 h of LPS stimulation (100 ng/mL). *n* = 3 biologically independent samples. NLRP3, **p = .0077 (WT+Con group vs. WT+LPS group), **p = .0026 (*IL‐10* ^−/−^+Con group vs. *IL‐10* ^−/−^+LPS group). Caspase‐1 p10, **p = .0046 (*IL‐10* ^−/−^+Con group vs. *IL‐10* ^−/−^+LPS group), **p = .0036 (WT+LPS group vs. *IL‐10* ^−/−^+LPS group), *p = .0344 (*IL‐10* ^−/−^+LPS group vs. *IL‐10* ^−/−^+rIL‐10+LPS group), *p = .0419 (WT+rIL‐10+LPS group vs. *IL‐10* ^−/−^+rIL‐10+LPS group). (**h, i)** Microplate reader assay exhibiting the effect of IL‐10 expression on the production of mitochondrial (**h**) and intracellular ROS (**i**) induced by LPS (100 ng/mL) in BMDMs. *n* = 6 biologically independent samples. (**h**) *p = .0122, ***p = .0005, ****p < .0001. (**i**) **p = .0044, ****p < .0001. **(j)** Confocal microscopy images showing the mitochondrial mass of BMDMs from each group using MitoTracker probe. Scale bars, 20 µm. (**k)** Immunoblotting and its quantitative analysis indicating the effect of IL‐10 on the expression of OPA1, MFN2, DRP1 and FIS1 for mitochondrial dynamics in BMDMs. *n* = 3 biologically independent samples. OPA1, *p = .0145 (WT+Con group vs. WT+LPS group), *p = .015 (*IL‐10* ^−/−^+Con group vs. *IL‐10* ^−/−^+LPS group), *p = .012. MFN2, **p = .0098, ***p = .0003, *p = .0159 (*IL‐10* ^−/−^+Con group vs. *IL‐10* ^−/−^+LPS group), *p = .0238 (*IL‐10* ^−/−^+LPS group vs. *IL‐10* ^−/−^+rIL‐10+LPS group). DRP1, **p = .0082, *p = .0358 (WT+LPS group vs. WT+rIL‐10+LPS group), *p = .0043 (*IL‐10* ^−/−^+LPS group vs. *IL‐10* ^−/−^+rIL‐10+LPS group), *p = .0157 (WT+LPS group vs. *IL‐10* ^−/−^+LPS group), *p = .0131 (WT +rIL‐10+LPS group vs. *IL‐10* ^−/−^+rIL‐10+LPS group), ****p < .0001. FIS1, ****p < .0001, **p = .0024, *p = .0332. Data are presented as mean  ±  SEM and analysed with a 95% confidence interval. *p* Values were calculated using one‐way ANOVA followed by Bonferroni's post hoc test.

Given that IL‐10, as a cytokine with anti‐inflammatory capabilities, plays a crucial role in regulating the immune response and, as previously mentioned, its activity can be regulated by CPT1A, we explored the role of IL‐10 in the anti‐inflammatory effects of CPT1A in the ALI model. WT mice and IL‐10 null mice were treated with LPS or PBS in combination, with rIL‐10 or PBS, followed by detailed testing after an interval of 24 h. The study finally showed that when the level of IL‐10 was reduced, airway inflammation was aggravated, especially the accumulation of macrophages and neutrophils in the inflammatory response triggered by LPS was enhanced. Accordingly, rIL‐10 supplementation was effective in slowing the growth of such inflammatory cells (Figure 4a). Local and systematic inflammation, manifested by IFN‐γ, IL‐6, G‐CSF and CXCL1/KC secretion in Balf and serum, was also aggravated by IL‐10 knockout and alleviated upon rIL‐10 treatment (Figure 4b and c). We also collected lung tissues for immunoblotting assay and histological staining. As shown in Figure 4d and e, IL‐10 deficiency, which further exacerbates the extent of damage to the ALI lung tissue. However, supplementation with rIL‐10 was effective in reversing the damage. The study revealed that endogenous IL‐10 production plays a maintenance role in inflammatory injury of ALI.

To explore the role of IL‐10 in the inflammatory response and mitochondrial stability of ALI macrophages, we isolated BMDMs from WT or *IL‐10* ^−/−^ mice and observed them for 24 h under LPS stimulation with or without rIL‐10 treatment as a variable. In the case of insufficient endogenous production of IL‐10, LPS significantly promoted the production of IL‐1β and IL‐6, two inflammatory mediators, whereas the addition of exogenous rIL‐10 effectively reduced their synthesis (Figure 4f). In parallel, BMDM studies revealed that depletion of IL‐10 accelerated LPS‐induced activation of the NLRP3/Caspase‐1 signalling pathway, while exogenous IL‐10 suppressed this inflammation reaction (Figure 4g). As for mitochondrial integrity and function, we discovered that IL‐10 deficiency exacerbated mitochondrial damage using MitoTracker fluorescent probe upon LPS challenge, which was rescued by rIL‐10 in BMDMs (Figure 4j). In addition, LPS‐triggered mitochondrial and intracellular ROS could also be increased by IL‐10 depletion, otherwise restrained by rIL‐10 administration (Figure 4h and i). When it came to mitochondrial dynamics, we demonstrated that lack of IL‐10 further reduced OPA1 expression while elevated DRP1 and FIS1 expression under LPS challenge; moreover, rIL‐10 treatment could partially reverse the changes (Figure 4k). Collectively, we speculate that IL‐10‐regulated mitochondrial homeostasis augments macrophage inflammatory effect in mice ALI model.

### 3.6 IL‐10 alters metabolic profiles and polarisation of macrophages upon LPS challenge

**FIGURE 5:**
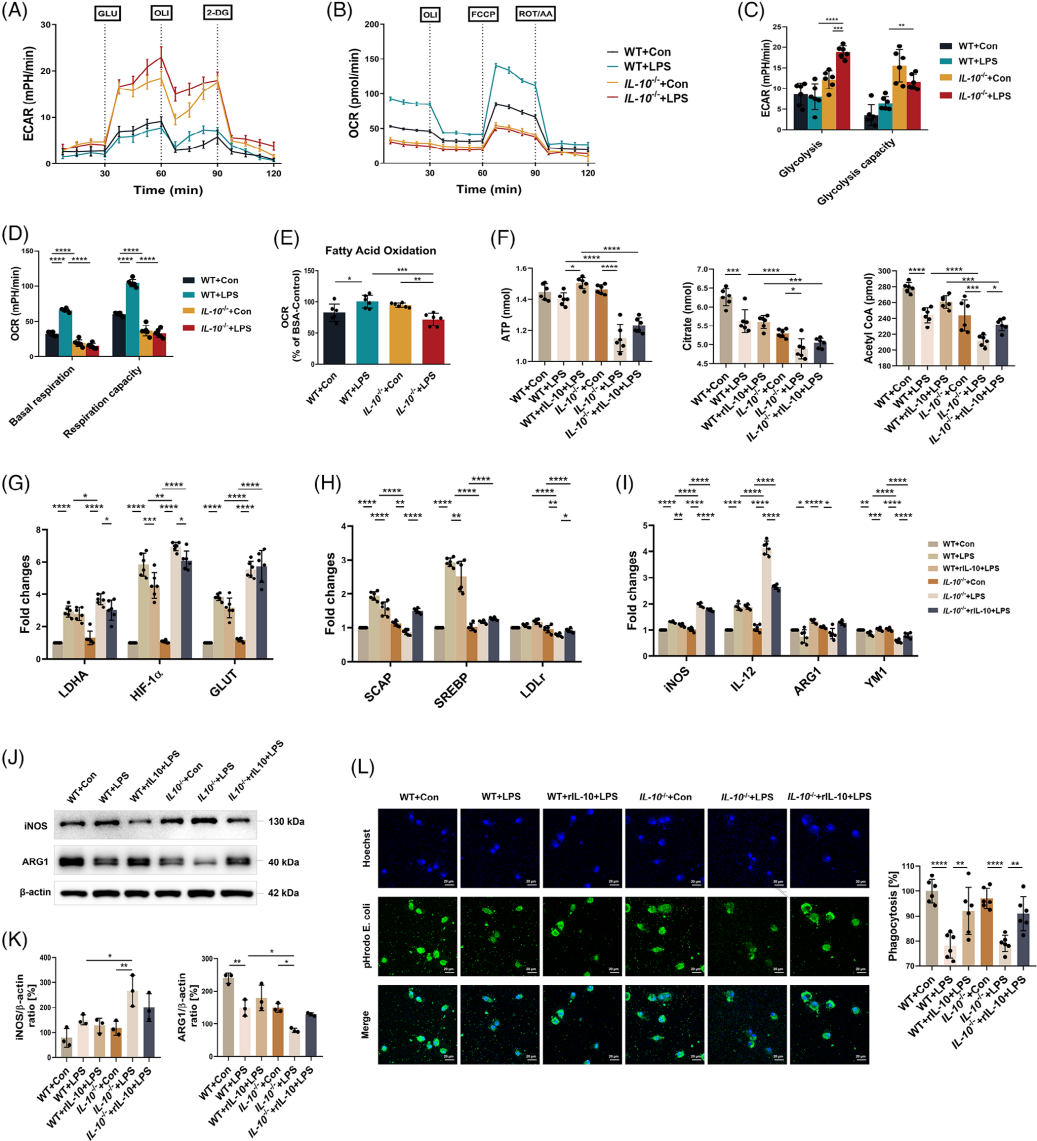
IL‐10 alters metabolic profiles and polarisation of macrophages upon LPS challenge. (**a, b)** ECAR (**a**) and OCR (**b**) of BMDMs from WT and *IL‐10* ^−/−^ mice were measured with a Seahorse XFe96 analyser. *n* = 6 biologically independent samples at each data point. (**c–e)** The glycolysis level and glycolysis capacity (**c**), as well as basal respiration and Respiration capacity (**d**), and FAO level (**e**) of BMDMs from WT and *IL‐10* ^−/−^ mice with or without LPS stimulation (100 ng/mL). *n* = 6 biologically independent samples. (**c**) ***p = .0004, **p = .0083, ****p < .0001. (**d**) ****p < .0001. (**e**) ***p = .0003, **p = .0029, *p = .0224. (**f)** After 12 h treatment with LPS (100 ng/mL) with or without rIL‐10 addition in BMDMs, generation of ATP, as well as intracellular FAO metabolites, citrate and acetyl CoA were measured. *n* = 6 biologically independent samples. *p = .0093, *p = .0145, ***p = .0006 (WT+rIL‐10+LPS group vs. *IL‐10* ^−/−^+rIL‐10+LPS group), ***p = .0001 (WT+Con group vs. WT+LPS group), *p = .017, ***p = .0004 (WT+rIL‐10+LPS group vs. *IL‐10* ^−/−^+rIL‐10+LPS group), ***p = .0001 (*IL‐10* ^−/−^+Con group vs. *IL‐10* ^−/−^+LPS group), ****p < .0001. (**g, h)** Induction of LDHA, HIF‐1α and GLUT (**g**), as well as SCAP, SREBP and LDLr (**h**) mRNA expression in WT and *IL‐10* ^−/−^ BMDMs was estimated by qRT‐PCR. Data are expressed as fold change. *n* = 6 biologically independent samples. (**g**) *p = .0407 (*IL‐10* ^−/−^+LPS group vs. *IL‐10* ^−/−^+rIL‐10+LPS group), *p = .0164 (WT+LPS group vs. *IL‐10* ^−/−^+LPS group), *p = .035, **p = .0038, ***p = .0008, ****p < .0001. (**h**) **p = .0034, **p = .0063, *p = .0202, **p = .0061,****p < .0001. (**i)** Induction of iNOS, IL‐12, ARG1 and YM1 mRNA expression in WT and *IL‐10* ^−/−^ BMDMs was analysed via qRT‐PCR. Data are expressed as fold change. *n* = 6 biologically independent samples. **p = .0018, *p = .0245, *p = .0286, **p = .0025, ***p = .0004, ****p < .0001. (**j, k)** Immunoblotting (**j**) and its quantitative analysis (**k**) verifying the effect of IL‐10 on iNOS and ARG1 expression for macrophage polarisation in BMDMs with or without LPS (100 ng/mL) challenge. *n* = 3 biologically independent samples. iNOS, *p = .0287, **p = .0052. ARG1, **p = .0011, *p = .011 (*IL‐10* ^−/−^+Con group vs. *IL‐10* ^−/−^+LPS group), *p = .0112 (WT+LPS group vs. *IL‐10* ^−/−^+LPS group). (**l)** The phagocytic capacity of BMDMs from WT and *IL‐10* ^−/−^ mice was evaluated using the pHrod Green *E. coli* BioParticles through both confocal microscopy (showed in the left panel; scale bars, 20 µm) and fluorescence plate reader (showed in the right panel). *n* = 6 biologically independent samples. **p = .002 (WT+LPS group vs. WT +rIL‐10+LPS group), **p = .0088 (*IL‐10* ^−/−^+LPS group vs. *IL‐10* ^−/−^+rIL‐10+LPS group), ****p < .0001. Data are presented as mean  ±  SEM and analysed with a 95% confidence interval. *p* Values were calculated using one‐way ANOVA followed by Bonferroni's post hoc test.

The changes of OCR and ECAR, as a measure of FAO‐OXPHOS and glycolysis were measured in WT and *IL‐10* ^−/−^ BMDMs stimulated by LPS. As shown in Figure 5a and c, *IL‐10* ^−/−^ BMDMs had higher ECAR basal levels and glycolytic capacity, indicating increased dependence on glycolysis when compared to WT BMDMs. Additionally, reduced basal OCR, ATP‐linked mitochondrial respiration and FAO‐coupled OCR were observed in *IL‐10* ^−/−^ BMDMs, suggesting the decreased predominance of FAO‐OXPHOS over glycolysis (Figure 5b, d and e). A decrease in acetyl CoA, citrate and ATP content was observed in IL‐10‐depleted BMDMs under LPS‐stimulated conditions. However, with the introduction of recombinant IL‐10, an increase in the level of acetyl CoA was observed, to levels similar to those of the WT phenotype (Figure 5f). By measuring the metabolism‐related gene signature of macrophages, we found that LPS stimulation dramatically increased the expression of glycolysis‐related genes LDHA, HIF‐1α, GLUT in WT and *IL‐10^−/‐^* BMDMs, while the exaggerated glycolysis induced by IL‐10 depletion could be partly alleviated by the addition of exogenous IL‐10 addition (Figure 5g). Meanwhile, the FAO‐associated genes LDLr and SCAP were downregulated in *IL‐10^−/‐^* BMDMs after LPS challenge, then was elevated by rIL‐10 treatment (Figure 5h). Overall, the results showed that IL‐10 inhibited LPS‐induced glycolysis and FAO‐OXPHOS in macrophages.

Subsequently, we assessed the expression of M1/M2 markers in WT and IL‐10‐deficient BMDMs after LPS stimulation to explore the role of IL‐10 in macrophage polarisation. As shown in Figure 5i, in comparison with the WT ones, BMDMs from *IL‐10* ^−/−^ mice showed an apparent increase in the M1 markers iNOS and IL‐12 and a reduce in the M2 markers ARG1 and YM1 after LPS challenge. Coherently, IL‐10 depletion also upregulated iNOS while inhibited ARG1 at protein expression level in LPS‐stimulated macrophages (Figure 5j and k). LPS‐impaired phagocytic capacity of macrophages could be recovered by rIL‐10 addition, though endogenous IL‐10 had no significant impact on this process. Our quantitative analysis results also indicate that the fluorescence intensity of LPS stimulation groups was significantly reduced compared with that of WT groups (Figure 5l). Notably, the above reactions could not be explained by cellular cytotoxicity resulted from IL‐10 defect and/or LPS challenge (Figure S8a and b). These data suggest that IL‐10 may inhibit aberrant inflammation in ALI by modulating the balance of macrophage polarisation.

### 3.7 Exogenous IL‐10 supplement confers protection against the aggravated ALI induced by CPT1A deficiency

**FIGURE 6:**
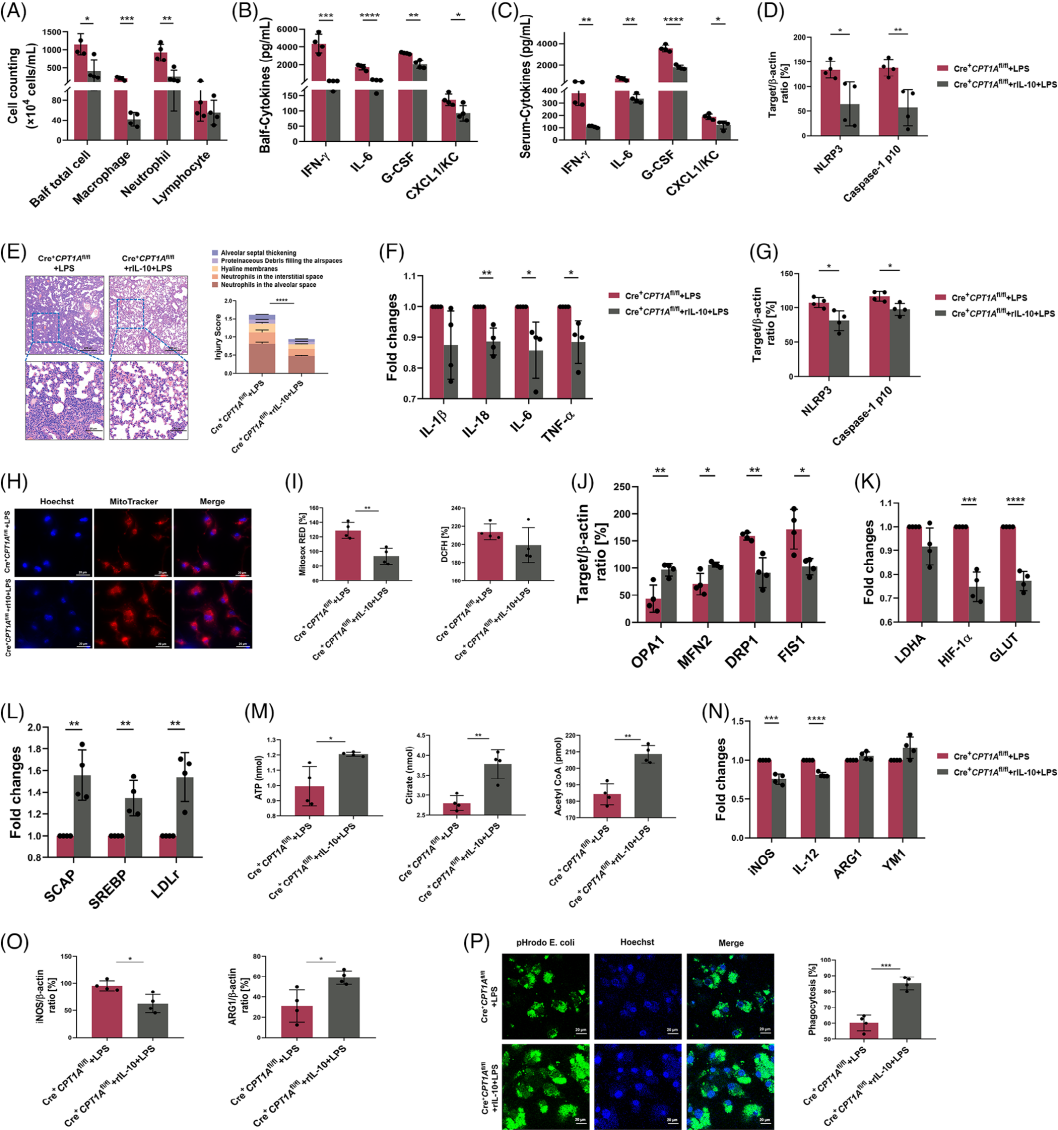
Exogenous IL‐10 supplement reverses the aggravated ALI, macrophages metabolic alterations and polarisation caused by CPT1A deficiency in LPS‐stimulated model. (a) Exogenous rIL‐10 (45 µg/kg) was administrated to Cre^+^ *CPT1A* ^fl/fl^ mice simultaneously with LPS (10 mg/kg) challenge. After 24 h, mice were euthanised and Balf was collected and estimated for total cell number, macrophage, neutrophil, and lymphocyte numbers. *n* = 4 biologically independent samples. *p = .0133, ***p = .0006, **p = .0041. (**b, c)** Production of cytokines IFN‐γ, IL‐6, G‐CSF, and CXCL1/KC in Balf and serum was assessed via ELISA kits. *n* = 4 biologically independent samples. (**b**) ***p = .0002, ****p < .0001, **p = .0011, *p = .031. (**c**) **p = .0013, **p = .0017, ****p < .0001, *p = .0144. (**d)** Quantitative immunoblotting analysis indicating the protective effect of rIL‐10 on the NLRP3/Caspase‐1 activation for lung inflammation in Cre^+^ *CPT1A* ^fl/fl^ mice. *n* = 4 biologically independent samples. *p = .0272, **p = .0072. (**e)** Representative images of H&E‐stained lung tissue sections of Cre^+^ *CPT1A* ^fl/fl^ mice after LPS challenge with or without rIL‐10 treatment. Scale bars in the upper panel, 200 µm; scale bars in the lower panel, 50 µm. *n* = 4 biologically independent samples. ****p < .0001. (**f)** After LPS stimulation for 6 h with or without rIL‐10 pretreatment, the mRNA expressions of cytokines IL‐1β, IL‐18, IL‐6 and TNF‐α in Cre^+^ *CPT1A* ^fl/fl^ BMDMs were examined by qRT‐PCR. Data are expressed as fold change. *n* = 4 biologically independent samples. **p = .0021, *p = .0206, *p = .0157. (**g)** Quantitative immunoblotting analysis showing the effect of rIL‐10 on the NLRP3/Caspase‐1 activation for macrophage inflammation after 24 h of LPS stimulation. *n* = 4 biologically independent samples. NLRP3, *p = .0166, Caspase‐1 p10, *p = .0145. (**h)** Confocal microscopy images showing the mitochondrial mass of BMDMs from each group using MitoTracker probe. Scale bars, 20 µm. (**i)** Microplate reader assay exhibiting the effect of rIL‐10 on mitochondrial and intracellular ROS production induced by LPS in Cre^+^ *CPT1A* ^fl/fl^ BMDMs. *n* = 4 biologically independent samples. **p = .004. (**j)** Quantitative immunoblotting analysis indicating the effect of rIL‐10 on the expression of OPA1, MFN2, DRP1 and FIS1 for mitochondrial dynamics in Cre^+^ *CPT1A* ^fl/fl^ BMDMs. *n* = 4 biologically independent samples. OPA1, **p = .0082. MFN2, *p = .0124. DRP1, **p = .0032. FIS1, *p = .013. (**k, l)** Induction of LDHA, HIF‐1α and GLUT (**k**), as well as SCAP, SREBP and LDLr (**l**) mRNA expression in Cre^+^ *CPT1A* ^fl/fl^ BMDMs with or without rIL‐10 addition was estimated by qRT‐PCR. Data are expressed as fold change. *n* = 4 biologically independent samples. (**k**) HIF‐1α, ***p = .0002, GLUT, ****p < .0001. (**l**) SCAP, **p = .0029, SREBP, **p = .0053, LDLr, **p = .0029. (**m)** After 12 h treatment with LPS with or without rIL‐10 supplement in BMDMs, production of ATP, as well as intracellular FAO metabolites, citrate and acetyl CoA were measured. *n* = 4 biologically independent samples. ATP, *p = .0173. citrate, **p = .0011. Acetyl CoA, **p = .003. (**n)** Induction of iNOS, IL‐12, ARG1 and YM1 mRNA expression in Cre^+^ *CPT1A* ^fl/fl^ BMDMs was analysed via qRT‐PCR. Data are expressed as fold change. *n* = 4 biologically independent samples. iNOS, ***p = .0003. IL‐12, ****p < .0001. (**o)** Quantitative immunoblotting analysis verifying the effect of rIL‐10 on iNOS and ARG1 expression for macrophage polarisation in Cre^+^ *CPT1A* ^fl/fl^ BMDMs upon LPS challenge. *n* = 4 biologically independent samples. iNOS, *p = .0142. ARG1, *p = .0179. (**p)** The phagocytic capacity of Cre^+^ *CPT1A* ^fl/fl^ BMDMs with or without rIL‐10 treatment was evaluated by the pHrod Green *E. coli* BioParticles through both confocal microscopy (showed in the upper panel; scale bars, 20 µm) and fluorescence plate reader (showed in the lower panel). *n* = 4 biologically independent samples. ***p = .0002. Data are presented as mean  ±  SEM and analysed with a 95% confidence interval. *p* Values were calculated using unpaired Student's *t*‐test.

To further clarify the role of IL‐10 in the protective effects of macrophage CPT1A in ALI models, we firstly supplemented Cre^+^ *CPT1A* ^fl/fl^ mice with exogenous IL‐10 together with LPS challenge. Results showed that Cre^+^ *CPT1A* ^fl/fl^ mice receiving LPS + rIL‐10 administration had less airway inflammatory cells, especially macrophages and neutrophils aggregation, as well as decreased IFN‐γ, IL‐6, G‐CSF, CXCL1/KC levels in Balf and serum, compared with Cre^+^ *CPT1A* ^fl/fl^ ALI mice without IL‐10 supplement (Figure 6a–c). Furthermore, rIL‐10 treatment inhibited inflammatory pathway activation (Figures S2a and 6d) and reduced diffuse damage in lungs of Cre^+^ *CPT1A* ^fl/fl^ ALI mice (Figure 6e). These data verify that aggravation of inflammatory damage caused by macrophage CPT1A deficiency within ALI lungs could be rescued by exogenous IL‐10.

We next asked whether the exacerbated inflammation in macrophages by CPT1A depletion could be alleviated by IL‐10 addition. As shown in Figure 6f, in the experimental model of Cre^+^ *CPT1A* ^fl/fl^ BMDMs, the overproduction of cytokines IL‐1β, IL‐18, IL‐6 and TNF‐α by LPS stimulation was effectively reduced by the introduction of exogenous IL‐10. In addition, with the presence of rIL‐10, the LPS‐induced inflammatory response was inhibited in Cre^+^ *CPT1A* ^fl/fl^ BMDM cells, especially the activation of the NLRP3/Caspase‐1 inflammatory pathway was suppressed (Figures S4a and 6g). These findings suggest that exogenous IL‐10 could compensate for excessive inflammation of macrophages caused by CPT1A deficiency.

To investigate whether the abnormal mitochondrial function described above in Cre^+^ *CPT1A* ^fl/fl^ BMDMs attributed to the lack of IL‐10, MitoTracker Red was used to stain the mitochondrial content of cells. The results showed that rIL‐10 treatment significantly restored the LPS‐induced decrease in fluorescence of Cre^+^ *CPT1A* ^fl/fl^ macrophages (Figure 6h). In line with the result, we proved that both levels of ROS were decreased in LPS‐stimulated Cre^+^ *CPT1A* ^fl/fl^ macrophages in the presence of IL‐10 by using mitochondria‐specific ROS indicator MitoSOX and intracellular ROS probe DCFH‐DA, demonstrating a potential positive effect of IL‐10 on mitochondrial ROS‐scavenging activity within Cre^+^ *CPT1A* ^fl/fl^ macrophages (Figure 6i). As for the study of mitochondrial dynamics, we found that the addition of rIL‐10 significantly increased the levels of OPA1 and MFN2, two mitochondrial fusion proteins, and effectively reduced the expression of DRP1 and FIS1 mitochondrial fission proteins in LPS‐induced Cre^+^ *CPT1A* ^fl/fl^ macrophages (Figures S4b and 6j). Collectively, IL‐10 addition in Cre^+^ *CPT1A* ^fl/fl^ BMDMs was able to restore the mitochondrial damage induced by CPT1A deficiency under LPS stimulation. Notably, compared with WT BMDMs, the CPT1A activator Lca could neither dampen inflammatory response (Figure S9a–c) nor improve mitochondrial dynamics (Figure S9d) in *IL‐10^−/−^* BMDMs, implying that IL‐10 acts as a crucial intermediate regulator for the protective effect of macrophage CPT1A on ALI/ARDS.

### 3.8 IL‐10 restores metabolic reprogramming and shifts polarisation caused by CPT1A deficiency in LPS‐stimulated macrophages

Because of the observed effects of IL‐10 on the aberrant macrophage functional status induced by CPT1A depletion, we investigated whether IL‐10 modulated metabolic changes during this process. As shown in Figure 6k and l, rIL‐10 addition significantly reduced the expression of glycolysis‐related genes HIF‐1α and GLUT, while upregulated FAO‐associated genes SCAP, SREBP and LDLr in Cre^+^ *CPT1A* ^fl/fl^ BMDMs upon LPS challenge, suggesting an increased FAO/glycolytic flux induced by IL‐10. Besides, levels of FAO intermediate metabolites acetyl CoA, citrate and ATP were all elevated by rIL‐10 administration in LPS‐stimulated Cre^+^ *CPT1A* ^fl/fl^ BMDMs (Figure 6m).

Regarding macrophage polarisation, we demonstrated that higher expression of M1 markers *iNOS* and *IL‐12*, and lower expression of M2 markers *ARG1* and *YM1* in Cre^+^ *CPT1A* ^fl/fl^ BMDMs after LPS stimulation could be partly reversed by rIL‐10 addition (Figure 6n). Meanwhile, the IL‐10‐induced decrement in iNOS/ARG1 protein level was also confirmed in LPS‐challenged Cre^+^ *CPT1A* ^fl/fl^ BMDMs (Figures S7a and 6o). Consistently, impaired phagocytic capacity in CPT1A‐deficient macrophages upon LPS treatment was improved by IL‐10 supplement (Figure 6p). Overall, these data suggest that the changes in metabolic profiles and macrophage polarisation caused by CPT1A depletion are at least partly attributed to CPT1A‐regulated IL‐10 expression in the ALI model.

## 4 DISCUSSION

**FIGURE 7:**
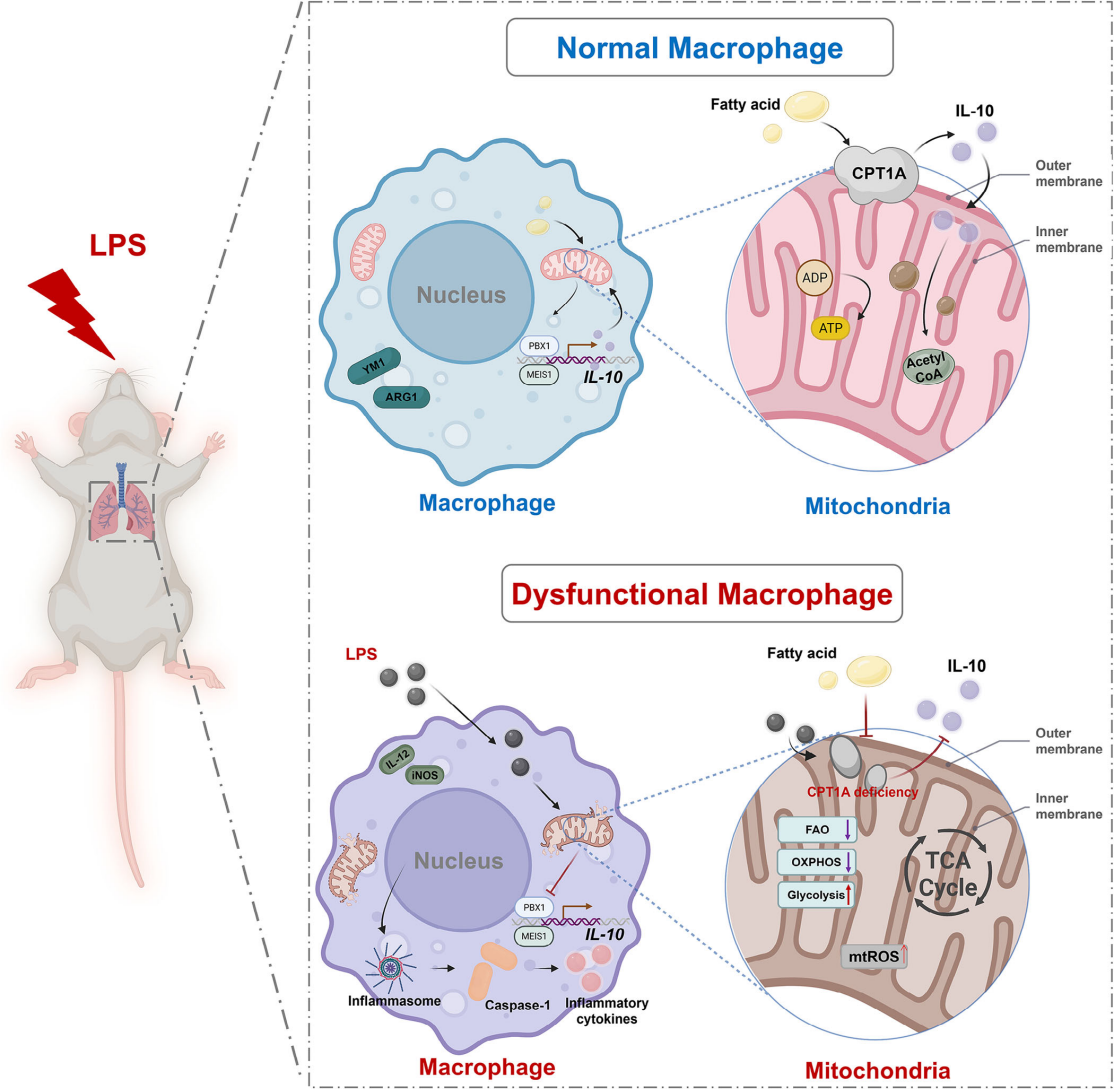
Illustration of the proposed mechanisms of the potent protective activity of CPT1A‐IL‐10 axis in LPS‐induced ALI mice. Some figure elements were created with BioRender.com.

In the late 19th century, Elias Metchnikoff first revealed the two main functions of macrophages: phagocytosis and elimination of microorganisms. Over the past few decades, macrophages have undergone evolution, and their functions have become increasingly sophisticated, widely involved in host defence, tissue stability and repair, pathology processes, and development. In order to manipulate these diverse functional pools, the innate immune system is managed by enabling a variety of activation states. Elaborate regulation of macrophage activation is essential for managing the inflammatory process and maintaining tissue homeostasis, especially in the therapy of diverse diseases associated with inflammation, such as ALI/ARDS. The process of lipid transformation plays a central role in macrophages’ physiological functions, which has a profound impact on the cell's biology, influencing cellular energy balance, the structure and composition of the cell membrane, and signalling processes.^9^ Regulation of fatty acid metabolism, such as the production and breakdown of fatty acids, is essential for the proper functioning of macrophages and the effectiveness of the overall immune system.^34^ Recently, the emerging field of ‘immunometabolism’ has been dedicated to unravelling the complex interactions between cellular metabolism and immune response, thereby contributing insights to the birth of innovative therapeutic regimens. The distinct metabolic profiles in activated macrophage are essential for appropriate immune cell function. Herein, we provided evidence that CPT1A, the key regulator of FAO, participated in metabolic alteration of activated macrophages and was able to modulate aberrant inflammation in the ALI model via upregulating IL‐10 production under physiological conditions, which could also maintain mitochondrial stability and drive the polarisation of macrophages into anti‐inflammatory direction. The defect of CPT1A‐IL‐10 axis interfered with macrophage metabolism, leading to the M1‐like macrophage polarisation upon LPS challenge, accompanied with aggravated inflammatory response and mitochondrial ROS (Figure 7). This study helps to reveal how macrophages regulate their immune and metabolic functions in disease States, thus pointing out a new possible direction for the development of therapies for ALI/ARDS.

Over the past decades, numerous studies have shown that FAO not only controls the bioenergy balance in macrophages, but also by generate certain metabolites to regulate signal transduction pathways and gene expression.^35^ The activity of fatty acid oxidation and fatty acid synthesis cycle has been monitored in M2‐differentiated macrophages, and these processes are closely related to cellular oxidative metabolism and ATP production.^36^ Oxidative metabolic processes, facilitated by FAO, can activate M2 macrophages through multiple pathways, involving the modulation of bioenergetics and the generation of nuclear and cytoplasmic Acyl‐CoA stores that serve as carbon sources for histone acetylation and stimulate the expression of genes that enhance the regulation of M2 macrophage functions such as fibrosis and tissue repair.^37^ However, some studies have cast doubt on the effectiveness of FAO in the anti‐inflammatory regulation of macrophage activation. Research has proven that the M2 polarisation process is not inhibited in macrophages deficient in the CPT2 gene.^14^ Therefore, further studies are essential to thoroughly unravel the effects of FAO on macrophage adaptation and characteristic transitions. In the present study, LPS challenge induced a more significant conversion of M1‐polarised macrophage with genetically deficient of CPT1A (Figure 3). Meanwhile, CPT1A‐deficient macrophages exhibited increased glycolysis and decreased FAO‐OXPHOS compared to the control ones (Figure 3). External stimuli often induce macrophages to consume more energy in a short time, when glycolysis becomes more active. At the same time, elevated glycolysis level and activated pentose phosphate pathway facilitate rapid ATP production and the synthesis of biosynthetic intermediates essential for generating proinflammatory factors. Meanwhile, the inhibition of OXPHOS results in the generation of substantial amounts of bactericidal ROS.^19^ Several literatures have focused on macrophage CPT1A expression and regarded it as a promising therapeutic strategy for inflammatory diseases.^16^ Other researchers pointed out that CPT1A inhibition could attenuate free FA‐induced inflammation in macrophages.^38^ These are consistent with our data showing that CPT1A protected against aberrant inflammation and maintained mitochondrial stability in macrophages when exposed to LPS (Figure 2), whereas CPT1A deficiency exacerbated pulmonary injury in an ALI mouse model (Figure 1). Therefore, CPT1A might be able to modify the metabolic process associated with inflammatory responses.

IL‐10 is a key immunomodulatory factor, which is produced by activated immune cells and plays a crucial part in controlling the process of excessive inflammatory response.^39^ IL‐10, whether endogenous or exogenous, has been shown to significantly mediate the inflammatory response in ALI mice^21^ and take part in the progression of immune cell recruitment, especially neutrophils,^40^, ^41^, ^42^ in multiple mouse ALI models, which is also proved in our previous findings.^21^ Ip et al.^20^ have found that IL‐10 could be involved in the regulation of inflammation‐induced changes in macrophage metabolic program. In this study, we observed that IL‐10 effectively blocked the metabolic transition of OXPHOS to glycolysis and supported the stability of mitochondrial structure during the LPS activated macrophages (Figures 4 and 5). Besides, IL‐10 was also found to enhance mitochondrial FAO and suppress macrophage polarisation towards M1 phenotype challenged with LPS (Figure 5), underscoring the pivotal role of IL‐10 in regulating the crosstalk between cell metabolism and immune function. In addition, our data suggest that CPT1A‐mediated fatty acid oxidation plays a protective role in macrophages during the ALI progression, which is largely regulated by IL‐10‐related signalling pathways. Exogenous IL‐10 supplementation to CPT1A‐deficient macrophages could effectively improve FAO level and decrease excessive inflammation caused by LPS (Figure 6), while CPT1A agonist (L‐carnitine) addition did not correct the aberrant inflammation and impaired mitochondrial dynamics in *IL‐10* ^−/−^ macrophages after LPS stimulation (Figure S9). Accordingly, we postulate that IL‐10 may be situated downstream of CPT1A, assuming the regulatory role in FAO and macrophage activation. The immune‐regulatory effects of IL‐10 hinges on the interactions with its receptor (IL‐10R), which is composed of a heterodimer of two subunits: high‐affinity IL‐10RA and low‐affinity IL‐10RB.^18^ Our data showed that CPT1A deficiency could not only inhibit the mRNA level and secretion of IL‐10, but also reduce the expression of its receptor IL‐10RA in LPS‐stimulated BMDMs (Figure 3). To address mechanistic links to IL‐10 gene modulation, we investigated the transcription factor necessary for IL‐10 expression in BMDMs, and discovered the existence of CPT1A culminated in activation of PBX1 and MEIS1 (Figure 3).^43^, ^44^ Therefore, we hypothesised that CPT1A‐predominant FAO at least partly depends on the capacity of IL‐10 production in macrophages. Although IL‐10 has previously been illustrated to take part in regulating cellular glycolysis, our study complements the gap in the role of CPT1A‐IL‐10 axis in immunoinflammatory modulation by elevating macrophage FAO level.

There are some shortcomings in the current academic exploration. We showed that the FAO rate‐limiting enzyme CPT1A suppressed the inflammatory responses of macrophages in an IL‐10‐dependent manner, leading to the alleviation of acute lung injury. However, the potential role of CPT1A in regulating other cell types in the lungs, and their contributions to the inflammatory response during the progression of ALI/ARDS, remains to be explored. A second limitation is that the underlying mechanism by which CPT1A regulates the expression of IL‐10/IL‐10RA in macrophages upon LPS exposure was not fully illustrated in this study. Additionally, the clinical relevance of CPT1A in acute pulmonary inflammatory diseases in humans needs to be validated using clinical biopsy, serum and Balf samples. Furthermore, the Lyz2‐cre system used to generate the conditional Cre^+^ *CPT1A* ^fl/fl^ mice is primarily expressed in mouse myeloid cells, such as neutrophils and monocytes in the bone marrow. This leads to the possibility that other myeloid cell lines are also affected in our mouse model. Last but not the least, IL‐10 has been illustrated could inhibit glucose uptake and glycolysis. However, according to our results that IL‐10 increased cellular ATP, citrate and acetyl‐CoA in macrophages lacking CPT1A, which were expected to already have limited mitochondrial FAO capacity. We deemed due to the reduced glycolysis mediated by IL‐10, which is expected to further constrain substrate (pyruvate) availability for mitochondrial metabolism; yet our data suggest that mitochondrial metabolism is increased – presumably due to enhanced amino acid catabolism, which is also crucial for maintaining proper immune cell function during the immune response. The alternatively activated progress of macrophage could lead to significant changes in amino acid catabolism, including arginine and proline metabolism.^45^ By contrast, classically activated M1‑type macrophages mainly rely on glutamine metabolism.^7^, ^46^ Therefore, changes in amino acid metabolism can affect the disease process by regulating the activation state of macrophages, which is also worthy of further investigation in the future.

In conclusion, we identified the reduced expression of CPT1A in macrophages of LPS‐induced ALI mouse model. By constructing mice harbouring a conditional depletion of macrophage CPT1A (Cre^+^ *CPT1A* ^fl/fl^), we intuitively observed the protective role of CPT1A in inhibiting neutrophil accumulation, and reducing lung inflammatory damage. By means of mouse BMDMs, CPT1A exhibited preeminent anti‐inflammatory and mitochondria‐regulatory effects on the cells challenged by LPS. Mechanistically, CPT1A could not only modulate metabolic reprogramming between glycolysis and FAO‐OXPHOS but also suppress macrophage polarisation towards M1 phenotype induced by LPS. Besides, CPT1A has capable of increasing the expression of IL‐10/IL‐10RA by activating the transcription factors. When lack of endogenous IL‐10, similar reactions occurred both in ALI mice and macrophages upon LPS stimulation in comparison with the Cre^+^ *CPT1A* ^fl/fl^ group. We further ascertained that the addition of exogenous IL‐10 to Cre^+^ *CPT1A* ^fl/fl^ macrophages effectively reversed the metabolic program associated with the inflammatory response caused by LPS. However, CPT1A agonist supplementation could not correct the aberrant inflammation and impaired mitochondrial dynamics in *IL‐10* ^−/−^ macrophages. Consequently, we anticipated that CPT1A‐IL‐10 axis, bridging cellular metabolism and immunological function in macrophages, may pave the way for new approaches to address inflammatory diseases, particularly ALI/ARDS, where effective treatments are currently lacking.

## AUTHOR CONTRIBUTIONS

Wei Gao, Kun Wang and Qiang Li are responsible for the content of the manuscript. They conceived and designed the study. Muyun Wang, Ximing Liao, Haiyang Hu, Di Wu, Jing Gao and Linlin Meng carry out experiments with technical guidance from Wei Gao, Qiang Li and Kun Wang, Feilong Wang gives guidance on the Seahorse experiments. Muyun Wang, Wujian Xu, Shaoyong Gao, Jing Hua and Yuanyuan Wang coordinated to analyse the data and write the manuscript. Wei Gao, Kun Wang and Qiang Li carefully revised the entire article. All authors read and approved the final manuscript.

## CONFLICT OF INTEREST STATEMENT

The authors declare no conflict of interests.

## ETHICS STATEMENT

All animal experiments were implemented under the guidelines of Shanghai Committee for Accreditation of Laboratory Animal, and the research protocol was approved by the Laboratory Animal Research Center Review Board of Tongji University (Permit Number: TJBB03721106) (Shanghai, China). All surgery was performed under pentobarbital sodium anaesthesia with efforts to minimise animal suffering.

## Supporting information

Supporting information

## Data Availability

The raw data of single‐cell RNA‐seq generated in this study have been deposited in Genome Sequence Archive with accession ID CRA008837. The remaining data that support the findings of this study are available from the corresponding author upon reasonable request.
