# Supplementary material for: CPT1A‐IL‐10‐mediated macrophage metabolic and phenotypic alterations ameliorate acute lung injury
Source: Clin Transl Med. 2024 Aug 1;14(8):e1785. doi: 10.1002/ctm2.1785 (PMC11294017; doi:10.1002/ctm2.1785)
Supplement: Supplementary file 1 — Supporting information [file CTM2-14-e1785-s001.docx]

Supporting information for

**CPT1A-IL-10-mediated macrophage metabolic and phenotypic alterations ameliorate acute lung injury**

Muyun Wang^1^^, #^, Di Wu^1, #^, Ximing Liao^1, #^, Haiyang Hu^2^, Jing Gao^1^, Linlin Meng^3^, Feilong Wang^1^, Wujian Xu^1^, Shaoyong Gao^1^, Jing Hua^1^, Yuanyuan Wang^1^, Qiang Li^1,*^, Kun Wang^1,*^, Wei Gao^1,*^

^#^ These authors contributed equally: Muyun Wang, Di Wu, Ximing Liao.

***** Correspondence to

Dr. Wei Gao* Department of Pulmonary and Critical Care Medicine, Shanghai East Hospital, School of Medicine, Tongji University, No.150 Jimo Road, Pudong, Shanghai, P.R. China.

E-mail: grace19881118@126.com

Dr. Kun Wang* Department of Pulmonary and Critical Care Medicine, Shanghai East Hospital, School of Medicine, Tongji University, No.150 Jimo Road, Pudong, Shanghai, P.R. China.

E-mail: Dr_Wangk@tongji.edu.cn

Prof. Qiang Li* Department of Pulmonary and Critical Care Medicine, Shanghai East Hospital, School of Medicine, Tongji University, No.150 Jimo Road, Pudong, Shanghai, P.R. China.

E-mail: liqressh1962@163.com

**SUPPLEMENTARY TABLE 1 The sequences of qRT-PCR primers used in this study.**

| **Primer** | **Sequence (5' to 3')** | **Species** |
| --- | --- | --- |
| LDHA forward | TGCGTGCTGGAGCCACT | Mouse |
| LDHA reverse | GCGAGGAGAAGCAGCGTG | Mouse |
| HIF1α forward | TGCTCATCAGTTGCCACTTC | Mouse |
| HIF1α reverse | TGGGCCATTTCTGTGTGTAA | Mouse |
| GLUT forward | GCTGTGCTTATGGGCTTCTC | Mouse |
| GLUT reverse | AGAGGCCACAAGTCTGCATT | Mouse |
| SCAP forward | ACTGGACTGAAGGCAGGTCAA | Mouse |
| SCAP reverse | GCCTCTAGTCTAGGTCCAAAGAGTTG | Mouse |
| SREBP forward | GCGCCAGGAGAACATGGT | Mouse |
| SREBP reverse | CGATGCCCTTCAGGAGCTT | Mouse |
| LDLr forward | CTGTGGGCTCCATAGGCTATCT | Mouse |
| LDLr reverse | GCGGTCCAGGGTCATCTTC | Mouse |
| IL-1β forward | GCAGTGGTTCGAGGCCTAAT | Mouse |
| IL-1β reverse | GCTGCTTCAGACACTTGCAC | Mouse |
| IL-6 forward | TAGTCCTTCCTACCCCAATTTCC | Mouse |
| IL-6 reverse | TTGGTCCTTAGCCACTCCTTC | Mouse |
| IL-18 forward | TCTTGGCCCAGGAACAATGG | Mouse |
| IL-18 reverse | CAGGCTGTCTTTTGTCAACGA | Mouse |
| TNF-α forward | CCACGTCGTAGCAAACCAC | Mouse |
| TNF-α reverse | TTGTCCCTTGAAGAGAACCTG | Mouse |
| iNOS forward | TGCCACGGACGAGACGGATAG | Mouse |
| iNOS reverse | CTCTTCAAGCACCTCCAGGAACG | Mouse |
| IL-12 forward | CAGAAAGGTGCGTTCCTCGTA | Mouse |
| IL-12 reverse | GCCCCTTTGCATTGG | Mouse |
| ARG1 forward | CTCCAAGCCAAAGTCCTTAGAG | Mouse |
| ARG1 reverse | AGGAGCTGTCATTAGGGACATC | Mouse |
| YM1 forward | AGAAGGGAGTTTCAAACCTGGT | Mouse |
| YM1 reverse | GTCTTGCTCATGTGTGTAAGTGA | Mouse |
| IL-10 forward | GCTCTTACTGACTGGCATGAG | Mouse |
| IL-10 reverse | CGCAGCTCTAGGAGCATGTG | Mouse |
| IL-10RA forward | CCCATTCCTCGTCACGATCTC | Mouse |
| IL-10RA reverse | TCAGACTGGTTTGGGATAGGTTT | Mouse |
| PBX1 forward | CAGCGGGTTCTTCCAGTTCTT | Mouse |
| PBX1 reverse | CGAGTCCGTCACTGTATCCTC | Mouse |
| MEIS1 forward | GCAAAGTATGCCAGGGGAGTA | Mouse |
| MEIS1 reverse | TCCTGTGTTAAGAACCGAGGG | Mouse |
| PRER1 forward | GGTGGTCACGGAGTTAAAAACA | Mouse |
| PRER1 reverse | TCGGCATCCATTGGGGTCT | Mouse |
| β-actin forward | AACAGTCCGCCTAGAAGCAC | Mouse |
| β-actin reverse | CGTTGACATCCGTAAAGACC | Mouse |


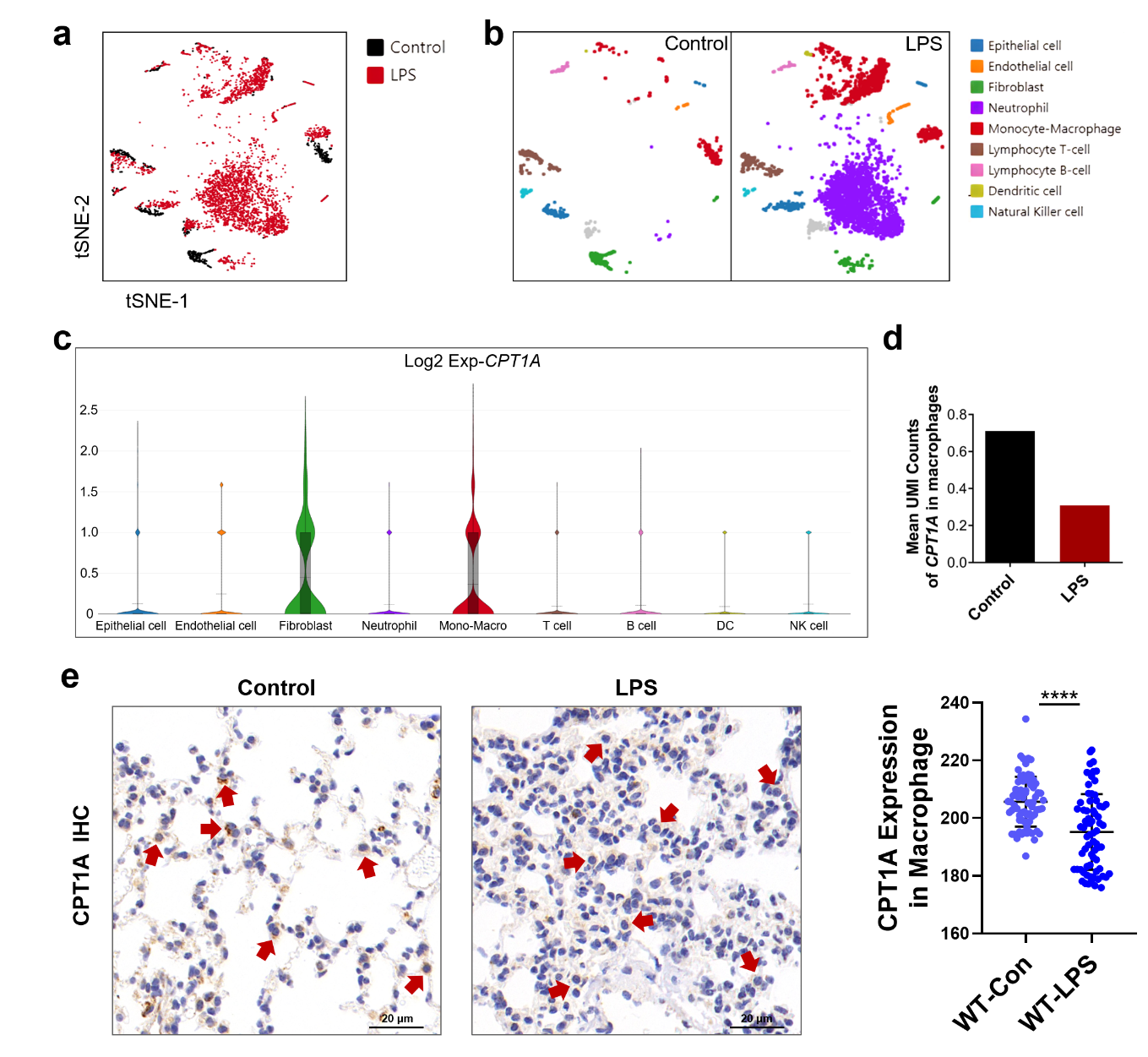


**SUPPLEMENTARY FIGURE 1 a** The t-SNE representation of aligned gene expression data in single cells collected from control (1,129 cells) and LPS-treated (4,478 cells) mouse lung samples showed cellular origins. **b** Unsupervised clustering identiﬁed 9 major pulmonary cell subsets. Each point depicts a single cell, colored based on the cluster designation. **c** Violin plots of CPT1A gene expression in single cells of each cell population (epithelial, endothelial, fibroblast, neutrophil, mono-macro, T, B, DC and NK cells) from lungs of control and LPS-treated mice. Boxes within violin plot show the median ± 1 quartile, with the whiskers extending from the hinge to the smallest or largest value within 1.5× interquartile range from the box boundaries. **d** The mean Unique Molecular Identiﬁer (UMI) counts per cell of *CPT1A* gene in macrophage cluster. **e** Immunohistochemical staining and quantitative analysis of CPT1A in lung samples of ALI mice. Scale bars, 20 µm. Red arrowheads indicate CPT1A-expressed macrophage within lungs. *n* = 70 each group from 6 biologically independent samples. *****p* < 0.0001. Data are presented as mean ± SEM and analyzed with a 95% confidence interval. *P* values were calculated using two-tailed unpaired Student t test.


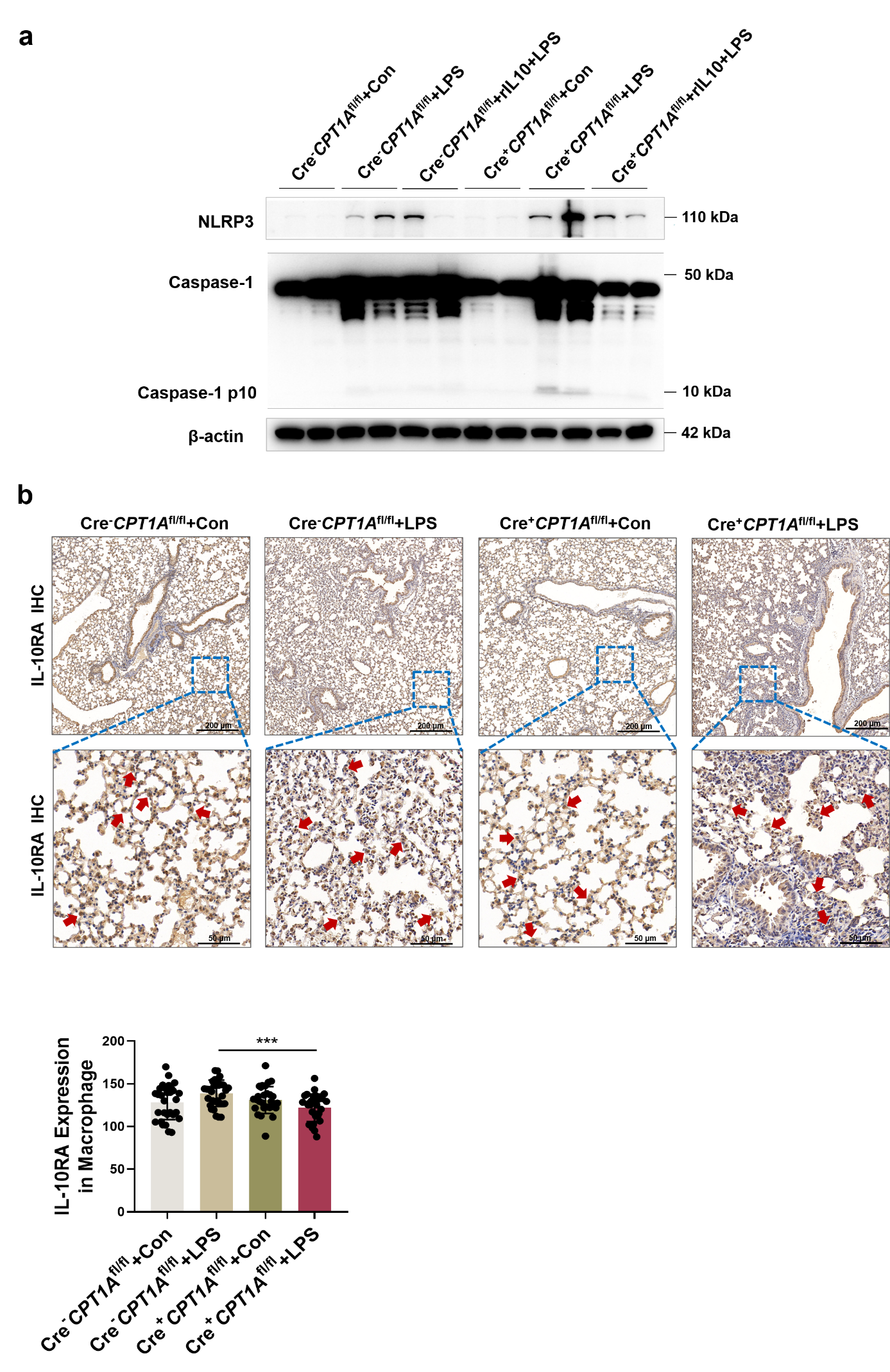


**SUPPLEMENTARY FIGURE 2 a** Immunoblotting assay demonstrating the regulatory effects of CPT1A-IL-10 on the activation of NLRP3/Caspase-1 pathway in lungs of ALI mice. **b** Immunohistochemical images of representative sections from mouse lungs stained with IL-10RA and quantitative analysis of IL-10RA protein levels in pulmonary macrophages from different groups. Red arrowheads indicate IL-10RA-expressed macrophage within mouse lungs. Scale bars in the upper panel, 200 µm; scale bars in the lower panel, 50 µm. *n* = 30 each group from 6 biologically independent samples. ****p* =0.0007. Data are presented as mean ± SEM and analyzed with a 95% confidence interval. *P* values were calculated using one-way ANOVA followed by Bonferroni's post hoc test.


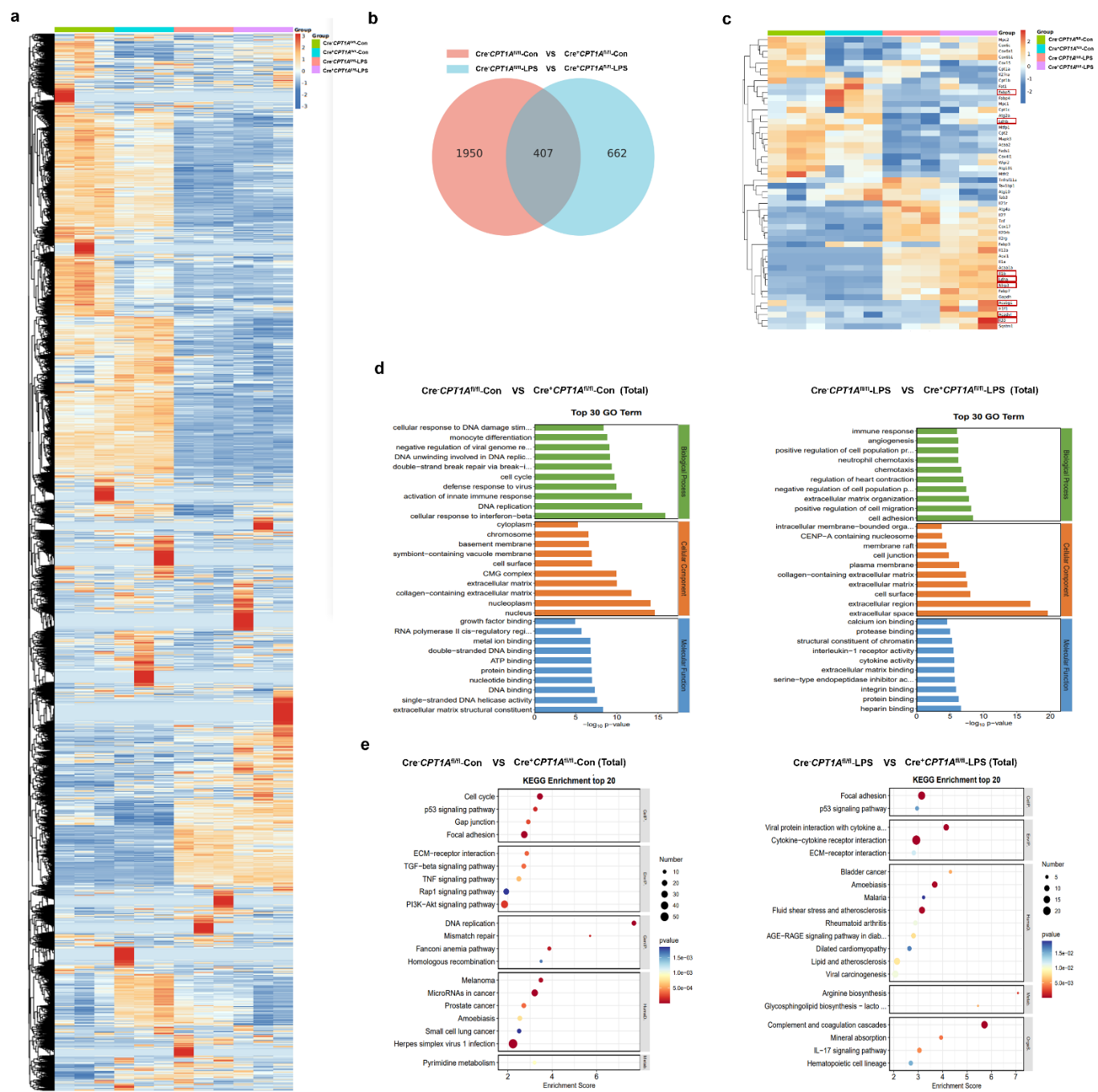


**SUPPLEMENTARY FIGURE 3 a**, Heat map of all differentially expressed genes in Cre^-^*CPT1A*^fl/fl^ and Cre^+^*CPT1A*^fl/fl^ BMDMs with or without LPS challenge. **b** Venn diagrams showing overlaps of differentially expressed genes among four experimental groups. **c** Heat map of the top 50 differentially-expressed gene among four groups. Red boxes highlight genes associated with cellular metabolism (*Fabp5*, *Ldhb, Ldha, Acsbg1, Acadvl*) and pro-inflammatory cytokine production (*Nlrp3*, *Il1b, Il33*). **d**, **e** Gene Ontology enrichment and Kyoto Encyclopedia of Genes and Genomes pathway analyses of Cre^-^*CPT1A*^fl/fl^ and Cre^+^*CPT1A*^fl/fl^ BMDMs with or without LPS stimulation.


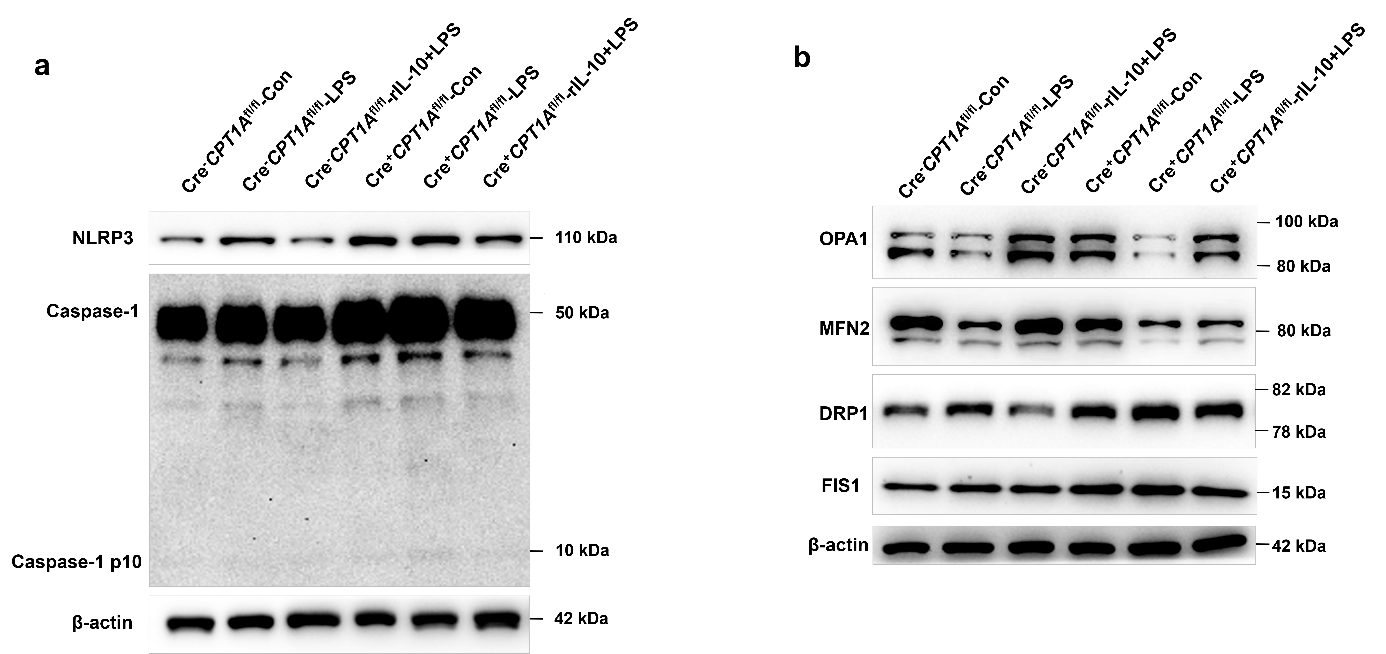


**SUPPLEMENTARY FIGURE 4** Immunoblotting assay demonstrating the regulatory effects of CPT1A-IL-10 on NLRP3/Caspase-1 p10 level for inflammasome activation (**a**), and OPA1, MFN2, DRP1, FIS1 level for mitochondrial dynamics (**b**) in BMDMs. *n* = 6 biologically independent samples.


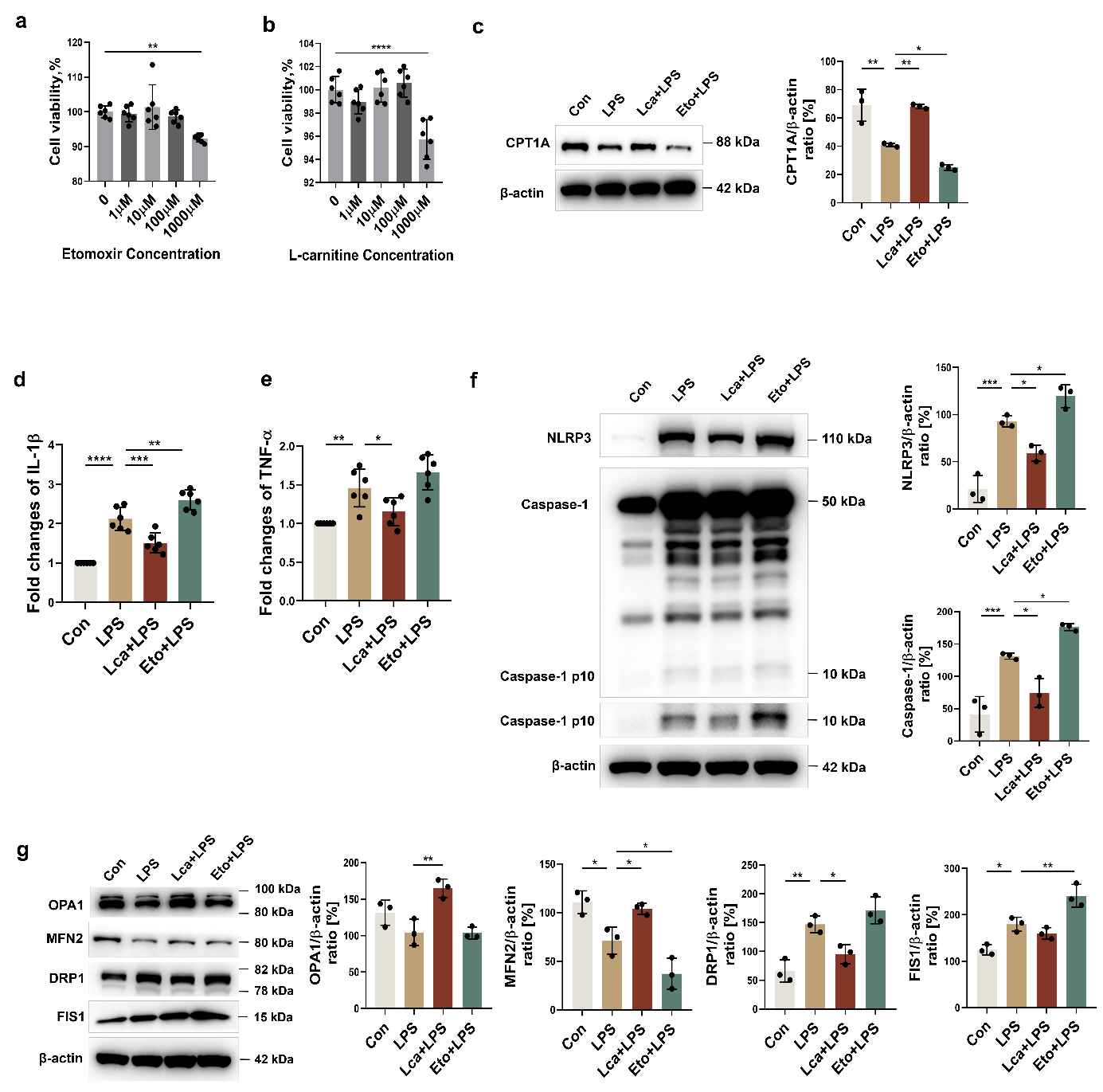


**SUPPLEMENTARY FIGURE 5 a, b** Effects of Etomoxir (Eto) and L-carnitine (Lca) at different concentrations on cell viability of BMDMs. *n* = 6 biologically independent samples. **a**, ***p* = 0.0016. **b**, *****p* < 0.0001. **c** Immunoblotting assay and quantitative analysis of CPT1A expression upon Lca (100 µM) or Eto (10 µM) treatment for 12 h. *n* = 3 biologically independent samples. ***p* = 0.0012 (Con group versus LPS group), ***p* = 0.0014, **p* = 0.0321. **d**, **e** Effects of Eto (10 µM) or Lca (100 µM) on LPS-induced IL-1β (**d**), and TNF-α (**e**) mRNA expression in BMDMs. Data are expressed as fold change. *n* = 6 biologically independent samples. **d**, ***p* = 0.005, ****p* = 0.0005, *****p* < 0.0001. **e**, ***p* = 0.0014, **p* = 0.0337. **f** Immunoblotting and its quantitative analysis indicating the effect of Eto (10 µM) or Lca (100 µM) on LPS-induced NLRP3/Caspase-1 pathway activation for macrophage inflammation. *n* = 3 biologically independent samples. NLRP3, ****p* = 0.0001, **p* = 0.0136 (LPS group versus Lca+LPS group), **p* = 0.0457. Caspase-1 p10, ****p* = 0.0009, **p* = 0.0147 (LPS group versus Lca+LPS group), **p* = 0.0482 (LPS group versus Eto+LPS group). **g** Immunoblotting assay and quantitative analysis verifying the effect of Eto (10 µM) or Lca (100 µM) on OPA1, MFN2, DRP1, FIS1 expression for mitochondrial dynamics in BMDMs under LPS stimulation. *n* = 6 biologically independent samples. OPA1, ***p* = 0.0029. MFN2, **p* = 0.0142 (Con group versus LPS group), **p* = 0.0357 (LPS group versus Lca+LPS group), **p* = 0.0315 (LPS group versus Eto+LPS group). DRP1, ***p* = 0.0021 (Con group versus LPS group), **p* = 0.0276. FIS1, **p* = 0.0115, ***p* = 0.0062. Data are presented as mean ± SEM and analyzed with a 95% confidence interval. *P* values were calculated using one-way ANOVA followed by Bonferroni's post hoc test.


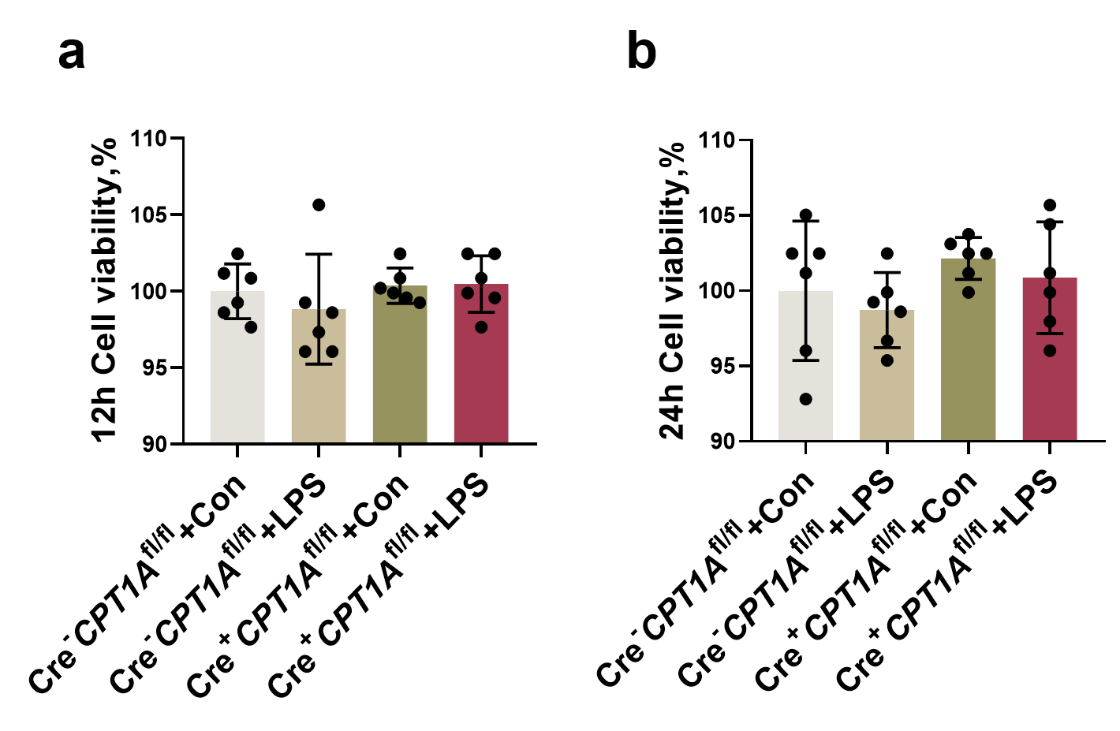


**SUPPLEMENTARY FIGURE 6** Cell viability of Cre^-^*CPT1A*^fl/fl^ and Cre^+^*CPT1A*^fl/fl^ BMDMs with or without LPS treatment for 12 h (**a**) and 24 h (**b**), respectively. *n* = 6 biologically independent samples. Data are presented as mean ± SEM and analyzed with a 95% confidence interval. *P* values were calculated using one-way ANOVA followed by Bonferroni's post hoc test.


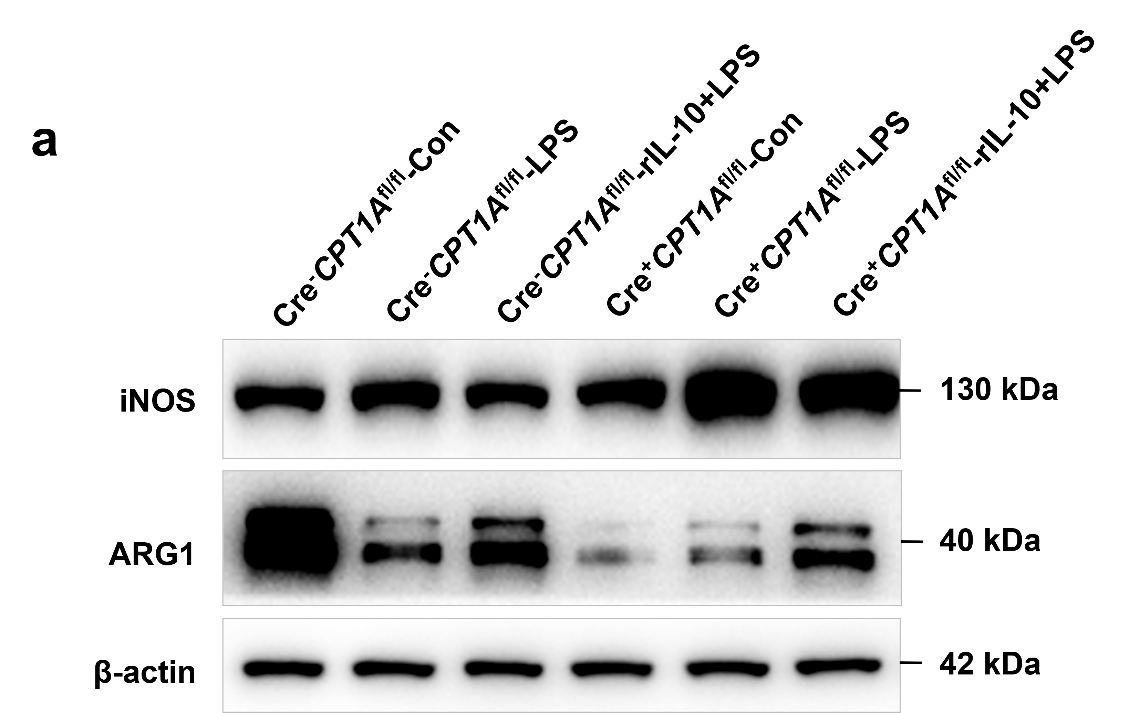


**SUPPLEMENTARY FIGURE 7** Immunoblotting assay demonstrating the regulatory effects of CPT1A-IL-10 on iNOS (**a**) and ARG1 (**b**) expression for macrophage polarization in BMDMs. *n* = 6 biologically independent samples.


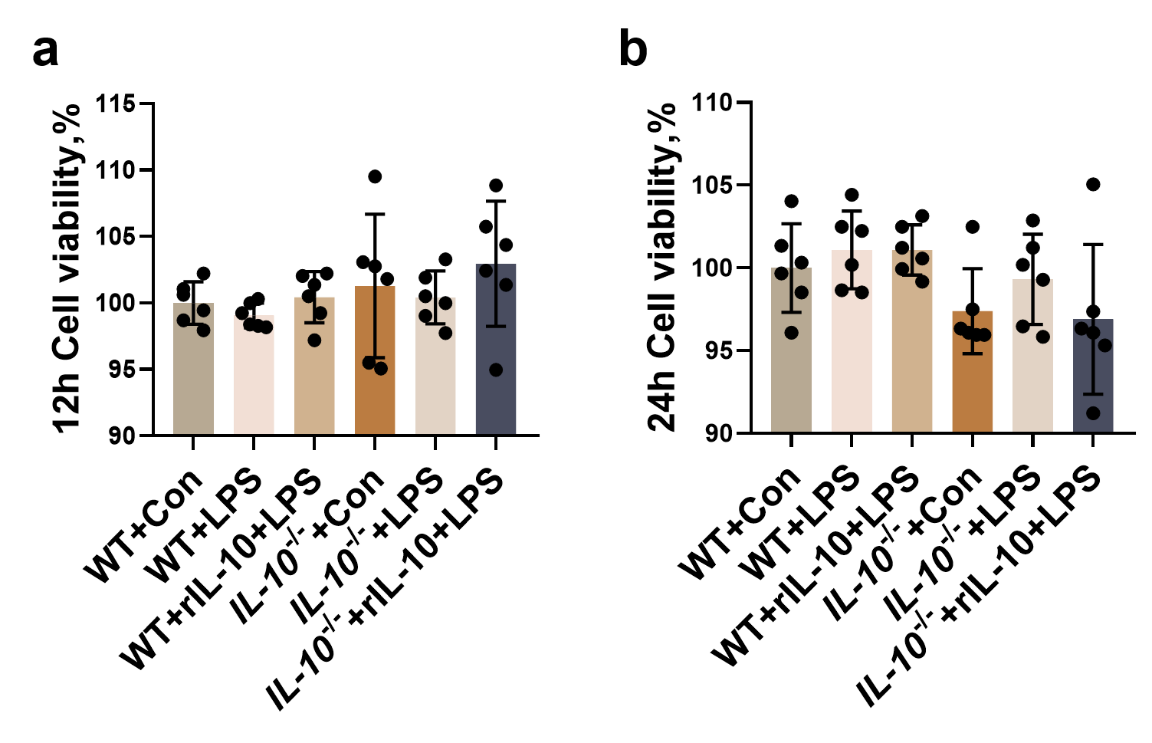


**SUPPLEMENTARY FIGURE 8** Effects of IL-10 level on the viability of BMDMs with or without LPS treatment for 12 h (**a**) and 24 h (**b**), respectively. *n* = 6 biologically independent samples. Data are presented as mean ± SEM and analyzed with a 95% confidence interval. *P* values were calculated using one-way ANOVA followed by Bonferroni's post hoc test.


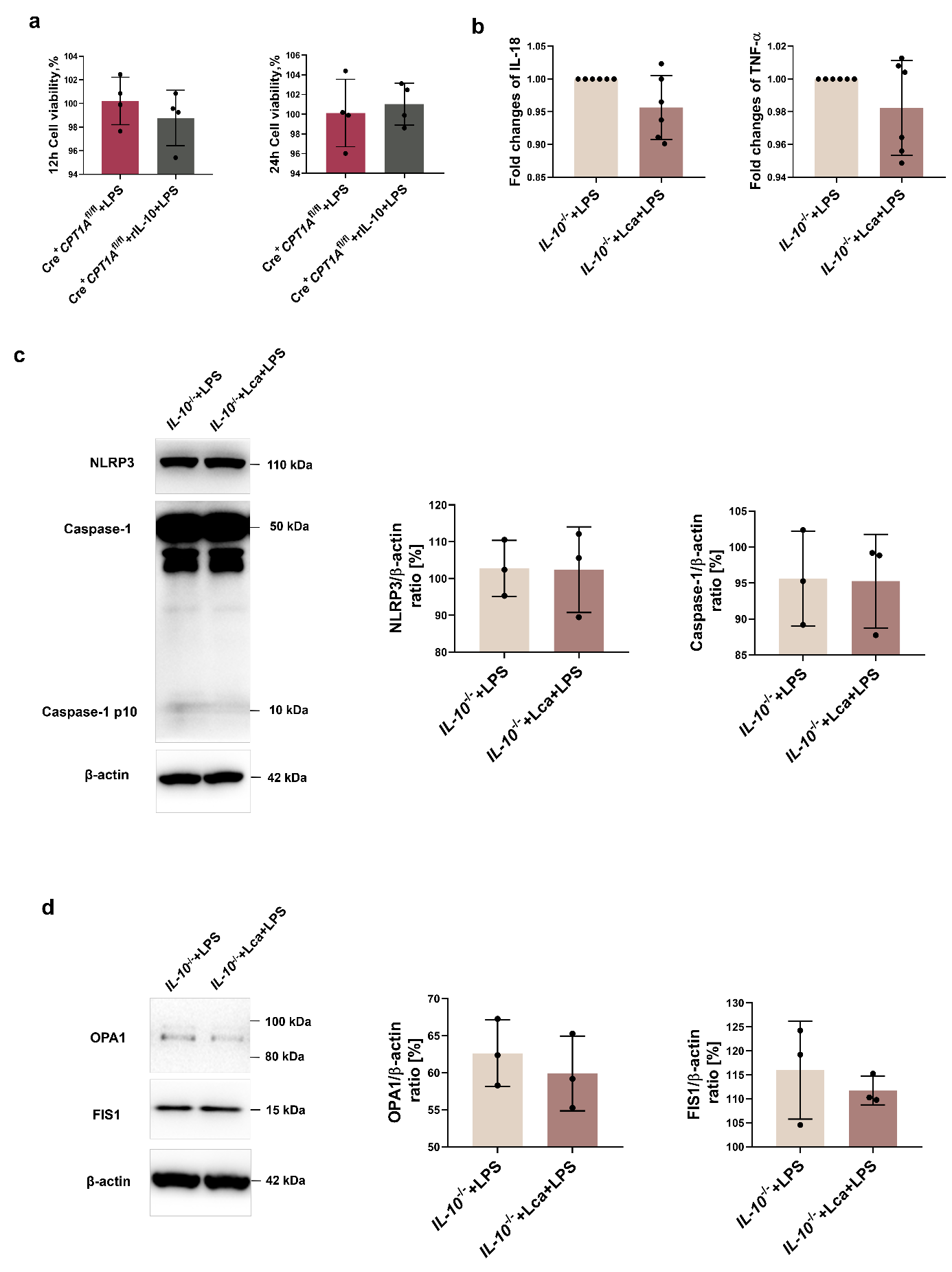


**SUPPLEMENTARY FIGURE 9 a** Effects of rIL-10 on the viability of Cre^+^*CPT1A*^fl/fl^ BMDMs with or without LPS treatment for 12 h and 24 h. *n* = 4 biologically independent samples. **b** Effects of Lca on LPS-induced IL-18 and TNF-α mRNA expression in IL-10^-/-^ BMDMs. Data are expressed as fold change. *n* = 4 biologically independent samples. **c, d** Immunoblotting assay and quantitative analysis exhibiting the effects of Lca on NLRP3/Caspase-1 pathway activation for macrophage inflammation (**c**), and OPA1, FIS1 expression for mitochondrial dynamics (**d**) in IL-10^-/-^ BMDMs under LPS stimulation. *n* = 3 biologically independent samples. Data are presented as mean ± SEM and analyzed with a 95% confidence interval. *P* values were calculated using two-tailed unpaired Student t test.

**ACKNOWLEDGEMENTS**

M.W. acknowledges the financial support of the China Scholarship Council program (File No. 202306260217).
